# ﻿The jumping plant-lice (Hemiptera, Psylloidea) in Urban Green Spaces of Bogotá (Colombia), with descriptions of two new species and redescription of *Mastigimascolombianus* Burckhardt, Queiroz and Drohojowska

**DOI:** 10.3897/zookeys.1209.117368

**Published:** 2024-08-08

**Authors:** Diana Isabel Rendón-Mera, Daniel Burckhardt, Juliana Durán, Valentina Ocampo, Sergio Andrés Vargas-Fonseca

**Affiliations:** 1 Naturhistorisches Museum, Augustinergasse 2, 4001 Basel, Switzerland Naturhistorisches Museum Basel Switzerland; 2 University of Basel, Petersplatz 1, 4001 Basel, Switzerland University of Basel Basel Switzerland; 3 Natural History Museum, Cromwell Road, London, UK Natural History Museum London United Kingdom; 4 Jardín Botánico de Bogotá José Celestino Mutis, Av. Calle 63 No. 68-95, Bogotá, D.C., Colombia Jardín Botánico de Bogotá José Celestino Mutis Bogotá Colombia; 5 Laboratorio de Entomología, Departamento de Biología, Pontificia Universidad Javeriana, Carrera 7, No. 43-82, Bogotá, D.C., Colombia Pontificia Universidad Javeriana Bogotá Colombia

**Keywords:** Biodiversity, city parks, insect–plant interactions, Neotropical region, psyllids, Sternorrhyncha, taxonomy, urbanisation

## Abstract

In a survey of the arthropod fauna of 33 Urban Green Spaces (UGS) in Bogotá, Colombia, between 2017 and 2019, 21 species (3,825 specimens) of Psylloidea were collected. These represent all seven recognised families of jumping plant-lice and include seven species identified only to genus. The specimens, all adults, were collected on 30 plant species used for arborization in the UGS. Two species are described as new (*Mastigimaslongicaudatus* Rendón-Mera, Burckhardt & Vargas-Fonseca, **sp. nov.** and *Leuronotaalbilinea* Rendón-Mera, Burckhardt & Vargas-Fonseca, **sp. nov.**), one species is redescribed (*Mastigimascolombianus* Burckhardt, Queiroz & Drohojowska) and one species is recorded for the first time from Colombia (*Calindatrinervis* Olivares & Burckhardt). Among the seven species identified only to genus is an undescribed species of *Melanastera*, representing a genus not previously known from Colombia. Fourteen species found during the survey are probably native (66%) and seven (33%) adventive. Our findings highlight the significance of UGS for preservation of biological diversity and stress the importance of using native plants in urban landscape planning for the conservation of the native entomofauna.

## ﻿Introduction

Urbanisation, the most irreversible form of land-use by the ever increasing human population, is one of the main drivers of the current extinction crisis (McKinney 2002; Seto et al. 2012; Díaz et al. 2019; Kong et al. 2021; Jaureguiberry et al. 2022). Accompanied by the degradation, fragmentation and loss of natural habitats (Foley et al. 2005; Elmqvist et al. 2013; Kong et al. 2021), urbanisation usually favours the presence of exotic species, leads to biotic homogeneity, and ultimately results in the loss of native species (McKinney 2002, 2008; Elmqvist et al. 2013; McDonald et al. 2018). Cities have dramatically expanded during the last decades and, as of today, more than half of the world’s population resides in urban areas with an expected increase to 70% by 2050 (Elmqvist et al. 2013; United Nations 2019).

As cities grow, Urban Green Spaces (UGS) become increasingly critical for supporting native organisms (Goddard et al. 2010; Aronson et al. 2014; Ives et al. 2016). These spaces comprise natural, semi-natural and artificial habitats, including remnants of native vegetation, parks, gardens, urban wastelands and green infrastructure (Tzoulas et al. 2007; Aronson et al. 2017; Lepczyk et al. 2017). However, not all UGS have equal conservation value, as the degree to which they can support biodiversity depends on several factors such as quality, size, connectivity, biotic interactions, land-use history and human population density (Aronson et al. 2017). Consequently, it is necessary to integrate ecological and biodiversity aspects into urban planning, to develop strategies for the design and management of these spaces to serve biodiversity conservation (McKinney 2002; Elmqvist et al. 2013; Aronson et al. 2017; McDonald et al. 2018).

Colombia is located in the north-west of South America and is one of the world’s megadiverse countries, home to approximately 10% of the world’s species and two of the world’s biodiversity hotspots: Tropical Andes and Tumbes–Chocó–Magdalena (Myers et al. 2000; Baptiste et al. 2017). At the same time, it is a highly urbanised country, with ~ 80% of its 50 million human inhabitants residing in urban areas (OECD 2022). This contrast is particularly evident in the Andean region, which exhibits both the highest levels of biological diversity and endemism, and of urbanisation and population density (Anselm et al. 2018; Carvajal-Castro et al. 2019). The Colombian capital Bogotá, the largest city in the country, is located in the middle of the Andes mountains, in the Eastern Ranges. Like other Latin American cities, much of Bogotá’s urban growth during the last two centuries has been unplanned and informal (Andrade et al. 2013), driven by an accelerated increase of rural-to-urban migration (Dufour and Piperata 2004). As a result, UGS only began to appear by the end of the 19^th^ century and, as late as the end of the 20^th^ century, became relevant under the concept of “Ecological Main Structure” (Andrade et al. 2013, 2014). Today, the concept has been decreed as one of the environmental determinants of land use-planning (Andrade et al. 2013, 2014). Bogotá has around 7,000 UGS of different scale and function, and ~ 1.4 million urban trees (Alcaldía Mayor de Bogotá 2009; Jardín Botánico de Bogotá 2023). However, despite the need for information on ecology and biodiversity to develop these strategies (McKinney 2002; Elmqvist et al. 2013; Aronson et al. 2017; McDonald et al. 2018), there are only a few studies that explore urban biodiversity in Colombia (e.g. Marín-Gómez et al. 2016; Ocampo Flórez et al. 2018; Durán-Prieto and Ocampo 2019; Durán-Prieto et al. 2020, 2023; Martínez and Morales 2020; Garizábal-Carmona and Mancera-Rodríguez 2021; Olaya-Arenas et al. 2022; Roncallo et al. 2022).

Psylloidea (jumping plant-lice or psyllids) constitute one of the superfamilies of Sternorrhyncha with more than 4,000 described and probably just as many undescribed species (Burckhardt et al. 2021; Ouvrard 2023). Psyllids are generally monophagous or narrowly oligophagous on one or a few closely related host plant species (Hodkinson 1974; Burckhardt et al. 2014; Ouvrard et al. 2015). A host plant is defined as that plant “on which a psyllid species completes its immature-to-adult life cycle” (Burckhardt et al. 2014). In practice, a host plant can be recognised by the presence of fifth instar immatures. Unlike the relatively immobile immatures, the winged adults disperse through flight or by air currents and are often found also on non-host plants (Burckhardt et al. 2014).

Psyllids are found in all biogeographic realms but are probably most species-rich in the tropics and the south temperate regions though these faunas are only poorly known, particularly those of the Afrotropical and Neotropical realms (Hollis 2004; Hodkinson 2009; Burckhardt and Queiroz 2020; Mauck et al. 2024). Little is known about the psyllid fauna of Colombia. Rendón-Mera et al. (2017) published a generic overview on the Colombian psyllids with a list of species known at the time. Additional information on psyllids from Bogotá is provided by Pinzón et al. (2002).

Here, the psyllids collected during a survey of the arthropod fauna of 33 UGS in Bogotá by the Botanical Garden “José Celestino Mutis” of Bogotá are discussed. The survey was conducted between 2017 and 2019, focussing on 30 species of native and exotic plants.

## ﻿Material and methods

### ﻿Material

**Figure 1.**
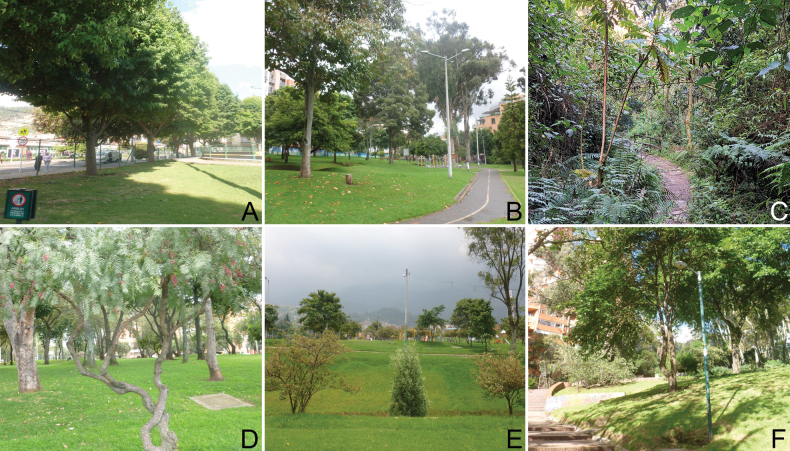
Some urban green spaces of Bogotá **A** Parque Altablanca **B** Parque Ginebra-Bella Suiza **C** Sendero Quebrada la Vieja **D** Parque La Francia **E** Parque San Cristóbal **F** Parque La Independencia.

**Figure 2.**
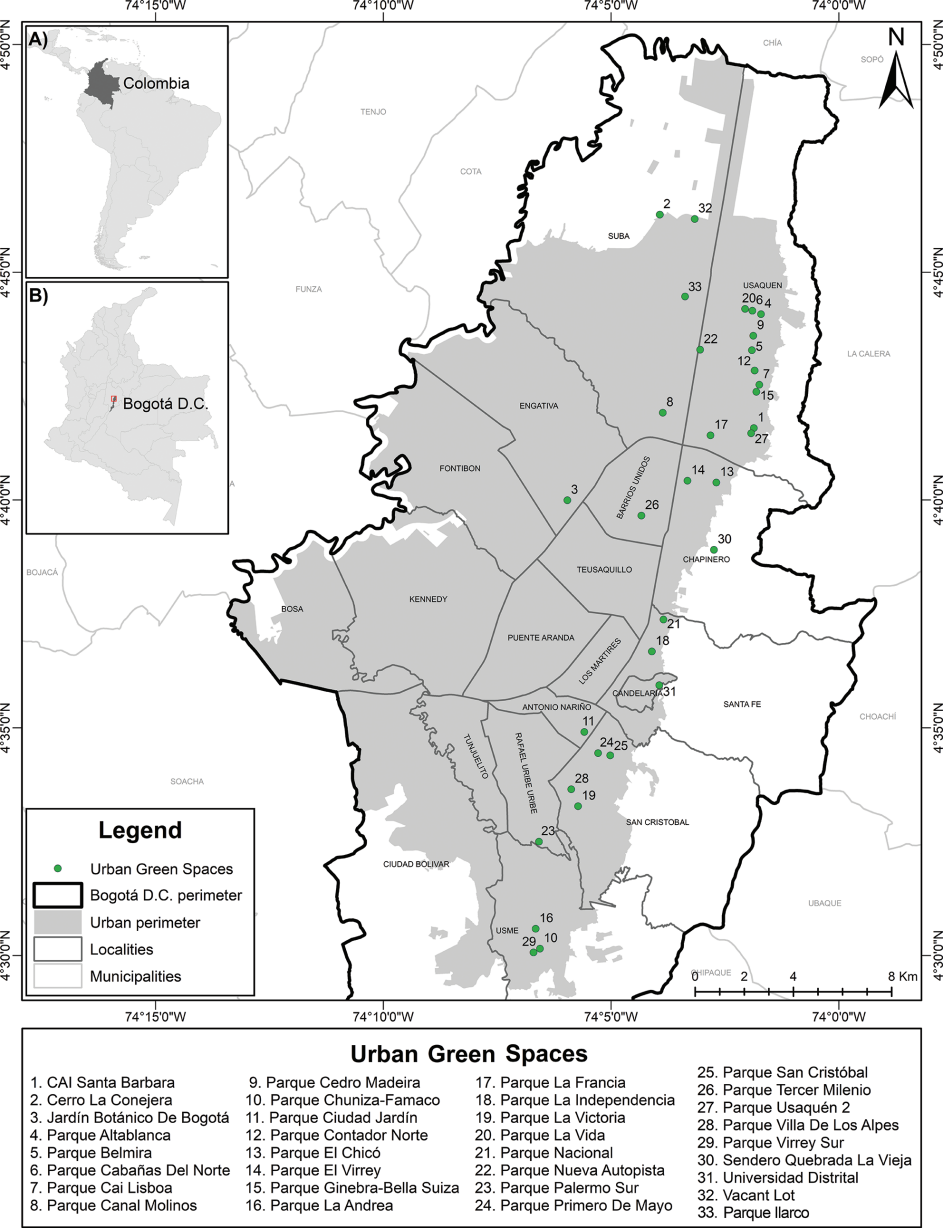
Map of Bogotá indicating localities and sampled urban green spaces.

Collections were conducted between 2017 and 2019 in 33 Urban Green Spaces (UGS) of nine of the 19 urban districts (“localidades”) of Bogotá (Figs 1, 2, Table 1, Appendix 1). Specimens were collected using sweep nets and entomological aspirators on the tree/shrub canopy cover of 30 plant species used for arborization in the city (Table 2). Unless stated otherwise, material is preserved pinned.

**Table 1.** Urban Green Spaces (UGS) with examined plants and psyllid species with number of collected adults. Plants confirmed in the literature as hosts or likely hosts are marked with § (see also text).

UGS	Plant species	Psyllid species	Number of adults
CAI Santa Barbara	*Quercushumboldtii* (Fagaceae)	*Calophyaschini*	1
Cerro La Conejera	§ *Acaciadealbata* (Fabaceae)	*Acizziaacaciaebaileyanae*	4
Cerro La Conejera	§ *Acaciamelanoxylon* (Fabaceae)	*Acizziauncatoides*	2
Cerro La Conejera	§ *Baccharis* sp. (Asteraceae)	*Calindagibbosa*	1
Cerro La Conejera	*Myrcianthesleucoxyla* (Myrtaceae)	*Tuthillialatipennis*	1
Jardín Botánico de Bogotá	*Myrcianthes* sp. (Myrtaceae)	*Trioza* sp. 1	1
Jardín Botánico de Bogotá	*Myrcianthes* sp. (Myrtaceae)	*Tuthillialatipennis*	1
Parque Altablanca	*Lafoensiaacuminata* (Lythraceae)	*Synozacornutiventris*	2
Parque Belmira	*Schinusareira* (Anacardiaceae)	*Acizziaacaciaebaileyanae*	1
Parque Belmira	§ *Schinusareira* (Anacardiaceae)	*Calophyaschini*	8
Parque Cabañas del Norte	*Lafoensiaacuminata* (Lythraceae)	*Acizziaacaciaebaileyanae*	5
Parque Cabañas del Norte	*Lafoensiaacuminata* (Lythraceae)	*Calophyaschini*	1
Parque Cabañas del Norte	*Lafoensiaacuminata* (Lythraceae)	*Ctenarytainaspatulata*	1
Parque Cabañas del Norte	*Lafoensiaacuminata* (Lythraceae)	*Mastigimascolombianus*	1
Parque Cabañas del Norte	*Pittosporumundulatum* (Pittosporaceae)	*Mastigimaslongicaudatus* Rendón-Mera, Burckhardt & Vargas-Fonseca, sp. nov.	1
Parque Cabañas del Norte	§ *Schinusareira* (Anacardiaceae)	*Calophyaschini*	419
Parque CAI Lisboa	*Pittosporumundulatum* (Pittosporaceae)	*Acizziauncatoides*	2
Parque CAI Lisboa	*Pittosporumundulatum* (Pittosporaceae)	*Synozacornutiventris*	6
Parque Canal Molinos	*Bocconiafrutescens* (Papaveraceae)	*Acizziaacaciaebaileyanae*	2
Parque Canal Molinos	§ *Cedrelamontana* (Meliaceae)	*Mastigimascolombianus*	17
Parque Canal Molinos	§ *Cedrelamontana* (Meliaceae)	*Mastigimaslongicaudatus* Rendón-Mera, Burckhardt & Vargas-Fonseca, sp. nov.	38
Parque Canal Molinos	§ *Ficus*americanasubsp.andicola (Moraceae)	*Synozacornutiventris*	9
Parque Cedro Madeira	§ *Ficus* sp. (Moraceae)	*Synozacornutiventris*	17
Parque Chuniza-Famaco	*Quercushumboldtii* (Fagaceae)	*Calophyaschini*	2
Parque Chuniza-Famaco	§ *Schinusareira* (Anacardiaceae)	*Calophyaschini*	558
Parque Ciudad Jardín	§ *Ficus* sp. (Moraceae)	*Synozacornutiventris*	9
Parque Ciudad Jardín	*Lafoensiaacuminata* (Lythraceae)	*Calophyaschini*	2
Parque Ciudad Jardín	*Lafoensiaacuminata* (Lythraceae)	*Syncoptozusmexicanus*	1
Parque Ciudad Jardín	§ *Schinusareira* (Anacardiaceae)	*Calophyaschini*	726
Parque Contador Norte	§ *Ficus* sp. (Moraceae)	*Synozacornutiventris*	117
Parque Contador Norte	*Lafoensiaacuminata* (Lythraceae)	*Acizziaacaciaebaileyanae*	2
Parque Contador Norte	*Liquidambarstyraciflua* (Altingiaceae)	*Acizziaacaciaebaileyanae*	2
Parque Contador Norte	*Liquidambarstyraciflua* (Altingiaceae)	*Calophyaschini*	1
Parque Contador Norte	*Liquidambarstyraciflua* (Altingiaceae)	*Ctenarytainaspatulata*	1
Parque Contador Norte	*Liquidambarstyraciflua* (Altingiaceae)	*Synozacornutiventris*	2
Parque Contador Norte	*Pittosporumundulatum* (Pittosporaceae)	*Synozacornutiventris*	1
Parque El Chicó	§ *Ficus* sp. (Moraceae)	*Synozacornutiventris*	10
Parque El Chicó	§ *Schinusareira* (Anacardiaceae)	*Calophyaschini*	7
Parque El Virrey	§ *Acaciamelanoxylon* (Fabaceae)	*Acizziauncatoides*	19
Parque El Virrey	§ *Cedrelamontana* (Meliaceae)	*Mastigimascolombianus*	38
Parque El Virrey	§ *Cedrelamontana* (Meliaceae)	*Mastigimaslongicaudatus* Rendón-Mera, Burckhardt & Vargas-Fonseca, sp. nov.	13
Parque El Virrey	*Crotoncoriaceus* (Euphorbiaceae)	*Calophyaschini*	14
Parque El Virrey	*Delostomaintegrifolium* (Bignoniaceae)	*Synozacornutiventris*	1
Parque El Virrey	*Feijoasellowiana* (Myrtaceae)	*Glycaspisbrimblecombei*	1
Parque El Virrey	*Fraxinuschinensis* (Oleaceae)	*Syncoptozusmexicanus*	1
Parque El Virrey	*Ligustrum* sp. (Oleaceae)	*Syncoptozusmexicanus*	2
Parque El Virrey	*Magnoliagrandiflora* (Magnoliaceae)	*Acizziauncatoides*	28
Parque El Virrey	§ *Magnoliagrandiflora* (Magnoliaceae)	*Syncoptozusmexicanus*	37
Parque El Virrey	*Pittosporumundulatum* (Pittosporaceae)	*Acizziauncatoides*	1
Parque El Virrey	*Salixhumboldtiana* (Salicaceae)	*Calophyaschini*	4
Parque Ginebra-Bella Suiza	*Ficus* sp. (Moraceae)	Triozidae gen. sp. 3	1
Parque Ginebra-Bella Suiza	*Liquidambarstyraciflua* (Altingiaceae)	*Mastigimaslongicaudatus* Rendón-Mera, Burckhardt & Vargas-Fonseca, sp. nov.	1
Parque Ginebra-Bella Suiza	§ *Schinusareira* (Anacardiaceae)	*Calophyaschini*	148
Parque Ginebra-Bella Suiza	*Schinusareira* (Anacardiaceae)	*Mastigimaslongicaudatus* Rendón-Mera, Burckhardt & Vargas-Fonseca, sp. nov.	2
Parque Ginebra-Bella Suiza	*Schinusareira* (Anacardiaceae)	*Synozacornutiventris*	1
Parque Tercer Ilarco	*Bocconiafrutescens* (Papaveraceae)	*Acizziaacaciaebaileyanae*	2
Parque Tercer Ilarco	*Clusia* sp. (Clusiaceae)	*Glycaspisbrimblecombei*	1
Parque Tercer Ilarco	*Crotoncoriaceus* (Euphorbiaceae)	*Calinda* sp.	1
Parque Tercer Ilarco	*Crotoncoriaceus* (Euphorbiaceae)	*Mastigimascolombianus*	1
Parque Tercer Ilarco	*Crotoncoriaceus* (Euphorbiaceae)	*Platycorypha* sp.	1
Parque Tercer Ilarco	*Crotoncoriaceus* (Euphorbiaceae)	*Synozacornutiventris*	1
Parque Tercer Ilarco	*Prunusserotina* (Rosaceae)	*Leuronotaalbilinea* Rendón-Mera, Burckhardt & Vargas-Fonseca, sp. nov.	1
Parque Tercer Ilarco	§ *Schinusareira* (Anacardiaceae)	*Calophyaschini*	8
Parque La Andrea	§ *Ficus* sp. (Moraceae)	*Synozacornutiventris*	99
Parque La Francia	*Lafoensiaacuminata* (Lythraceae)	*Synozacornutiventris*	2
Parque La Francia	§ *Schinusareira* (Anacardiaceae)	*Calophyaschini*	7
Parque La Independencia	§ *Ficus* sp. (Moraceae)	*Synozacornutiventris*	30
Parque La Independencia	*Liquidambarstyraciflua* (Altingiaceae)	*Synozacornutiventris*	1
Parque La Independencia	*Quercushumboldtii* (Fagaceae)	*Synozacornutiventris*	1
Parque La Victoria	*Pittosporumundulatum* (Pittosporaceae)	*Calophyaschini*	1
Parque La Victoria	*Quercushumboldtii* (Fagaceae)	*Calophyaschini*	5
Parque La Victoria	§ *Schinusareira* (Anacardiaceae)	*Calophyaschini*	250
Parque La Vida	§ *Ficus* sp. (Moraceae)	*Synozacornutiventris*	244
Parque La Vida	*Ficus* sp. (Moraceae)	Triozidae gen. sp. 3	1
Parque La Vida	*Pittosporumundulatum* (Pittosporaceae)	*Synozacornutiventris*	6
Parque Nacional	§ *Ficus* sp. (Moraceae)	*Synozacornutiventris*	4
Parque Nueva Autopista	*Ficus* sp. (Moraceae)	*Calophyaschini*	1
Parque Nueva Autopista	§ *Ficus* sp. (Moraceae)	*Synozacornutiventris*	3
Parque Palermo Sur	§ *Ficus* sp. (Moraceae)	*Synozacornutiventris*	2
Parque Palermo Sur	*Pittosporumundulatum* (Pittosporaceae)	*Acizziauncatoides*	1
Parque Palermo Sur	*Pittosporumundulatum* (Pittosporaceae)	*Calophyaschini*	1
Parque Palermo Sur	*Pittosporumundulatum* (Pittosporaceae)	Triozidae gen. sp. 2	1
Parque Palermo Sur	§ *Schinusareira* (Anacardiaceae)	*Calophyaschini*	26
Parque Primero de Mayo	§ *Ficus* sp. (Moraceae)	*Synozacornutiventris*	106
Parque Primero de Mayo	*Quercushumboldtii* (Fagaceae)	*Synozacornutiventris*	1
Parque San Cristóbal	*Lafoensiaacuminata* (Lythraceae)	*Synozacornutiventris*	1
Parque San Cristóbal	*Pittosporumundulatum* (Pittosporaceae)	*Synozacornutiventris*	1
Parque San Cristóbal	§ *Schinusareira* (Anacardiaceae)	*Calophyaschini*	89
Parque Tercer Milenio	§ *Clusia* sp. (Clusiaceae)	*Leuronotaalbilinea* Rendón-Mera, Burckhardt & Vargas-Fonseca, sp. nov.	85
Parque Tercer Milenio	*Magnoliagrandiflora* (Magnoliaceae)	*Calophyaschini*	6
Parque Tercer Milenio	§ *Magnoliagrandiflora* (Magnoliaceae)	*Syncoptozusmexicanus*	33
Parque Tercer Milenio	*Sambucusnigra* (Viburnaceae)	*Mastigimaslongicaudatus* Rendón-Mera, Burckhardt & Vargas-Fonseca, sp. nov.	2
Parque Usaquén 2	*Ficus* sp. (Moraceae)	*Calophyaschini*	1
Parque Usaquén 2	§ *Ficus* sp. (Moraceae)	*Synozacornutiventris*	4
Parque Usaquén 2	*Liquidambarstyraciflua* (Altingiaceae)	*Calophyaschini*	1
Parque Usaquén 2	*Pittosporumundulatum* (Pittosporaceae)	*Calophyaschini*	1
Parque Usaquén 2	*Pittosporumundulatum* (Pittosporaceae)	*Synozacornutiventris*	1
Parque Villa de los Alpes	§ *Ficus* sp. (Moraceae)	*Synozacornutiventris*	120
Parque Villa de los Alpes	§ *Schinusareira* (Anacardiaceae)	*Calophyaschini*	81
Parque Virrey Sur	§ *Ficus* sp. (Moraceae)	*Synozacornutiventris*	29
Parque Virrey Sur	§ *Schinusareira* (Anacardiaceae)	*Calophyaschini*	51
Sendero Quebrada La Vieja	*Miconiaelaeoides* (Melastomataceae)	*Ctenarytainaspatulata*	1
Sendero Quebrada La Vieja	*Miconiaelaeoides* (Melastomataceae)	*Melanastera* sp.	1
Sendero Quebrada La Vieja	*Piperbogotense* (Piperaceae)	*Acizziauncatoides*	1
Sendero Quebrada La Vieja	*Piperbogotense* (Piperaceae)	*Ctenarytainaeucalypti*	5
Universidad Distrital	*Acaciadecurrens* (Fabaceae)	*Acizziauncatoides*	15
Universidad Distrital	§ *Acaciamelanoxylon* (Fabaceae)	*Acizziauncatoides*	125
Universidad Distrital	§ *Baccharislatifolia* (Asteraceae)	*Calindagibbosa*	3
Universidad Distrital	*Baccharislatifolia* (Asteraceae)	*Calindatrinervis*	1
Universidad Distrital	*Crotoncoriaceus* (Euphorbiaceae)	*Acizziauncatoides*	2
Universidad Distrital	Lyciantheslycioides (Solanaceae)	*Acizziauncatoides*	24
Universidad Distrital	*Oreopanaxincisus* (Araliaceae)	*Acizziauncatoides*	1
Universidad Distrital	*Oreopanaxincisus* (Araliaceae)	*Ctenarytainaspatulata*	7
Universidad Distrital	*Quercushumboldtii* (Fagaceae)	*Acizziauncatoides*	1
Universidad Distrital	*Quercushumboldtii* (Fagaceae)	*Calindagibbosa*	1
Universidad Distrital	*Quercushumboldtii* (Fagaceae)	*Ctenarytainaspatulata*	1
Universidad Distrital	*Quercushumboldtii* (Fagaceae)	Triozidae gen. sp. 3	1
vacant lot	*Quercushumboldtii* (Fagaceae)	*Acizziauncatoides*	1
vacant lot	*Quercushumboldtii* (Fagaceae)	Triozidae gen. sp. 1	1

**Table 2.** Psyllid species, hosts (cf. text) and numbers of adult psyllid specimens collected on hosts and non-hosts.

Psyllid species	Host taxon	Adults on host	Adults on non-host
*Calophyaschini*	*Schinusareira*	2378	42 (= 1.8%)
*Synozacornutiventris*	*Ficus* spp.	803	28 (= 3.5%)
*Acizziauncatoides*	mimosoid Fabaceae	161	62 (= 38.5%)
*Leuronotaalbilinea* Rendón-Mera, Burckhardt & Vargas-Fonseca, sp. nov.	*Clusia* sp.	85	1 (= 1.2%)
*Mastigimascolombianus*	*Cedrelamontana*	55	2 (= 3.6%)
*Mastigimaslongicaudatus* Rendón-Mera, Burckhardt & Vargas-Fonseca, sp. nov.	*Cedrelamontana*	51	6 (= 11.8%)
*Syncoptozusmexicanus*	*Magnoliagrandiflora*	30	4 (= 13.3%)
*Acizziaacaciaebaileyanae*	mimosoid Fabaceae	4	12
*Calindagibbosa*	*Baccharis* spp.	4	1
*Ctenarytainaspatulata*	*Eucalyptus* spp.		11
*Ctenarytainaeucalypti*	*Eucalyptus* spp.		5
Triozidae gen. sp. 3	unknown		3
*Glycaspisbrimblecombei*	*Eucalyptus* spp.		2
*Tuthillialatipennis*	*Myrcianthes* spp.		2
*Calindatrinervis*	unknown		1
*Calinda* sp.	unknown		1
*Melanastera* sp.	unknown		1
*Platycorypha* sp.	unknown		1
*Trioza* sp. 1	unknown		1
Triozidae gen. sp. 1	unknown		1
Triozidae gen. sp. 2	unknown		1

Holotypes are deposited in the entomological collection of the Museo Javeriano de Historia Natural of the Pontificia Universidad Javeriana, Bogotá, Colombia (**MPUJ_ENT**). Paratypes and non-type material are deposited in MPUJ_ENT and the Naturhistorisches Museum, Basel, Switzerland (**NHMB**).

### ﻿Species description

Morphological terminology follows Bastin et al. (2023). Body length was taken from ethanol-preserved specimens in lateral view, measuring the distance from the tip of genal process to the tip of wings when folded over the body. All other measurements were taken from slide mounted specimens as indicated in Bastin et al. (2023). In *Leuronota*, vein length is measured as a linear distance. Measurements are given in mm and expressed as range (mean ± standard deviation). Slide preparation protocol follows Queiroz et al. (2017).

### ﻿Conventions

Taxa are arranged alphabetically (families and genera) following the classification of Burckhardt et al. (2021). Plant names and information of their origin correspond to POWO (2023). The following markings are used: (*) for new species records for Colombia and (‡) for adventive species. Material examined is presented per urban district, written in bold and arranged alphabetically. Plants mentioned in this section are those from which specimens were collected and not necessarily host plants as defined by Burckhardt et al. (2014). Distribution in Colombia is presented by department.

### ﻿Host plants

No immature psyllids were collected during the survey and none of the sampled plant species could, therefore, be confirmed as host in the sense of Burckhardt et al. (2014). Under “Host plant” we cite reliable literature records with the respective reference, or we discuss reasons for assuming that a particular plant constitutes a host. In Table 1 we use this information to classify plants into hosts (marked with §) and non-hosts.

### ﻿Abbreviations

**AL**—Antenna length; **AP**—Apical portion of female proctiger length; **BL**—Body length; **CRL**—Circumanal ring length; **DL**—Distal segment of aedeagus length; **FL**—Forewing length; **FP**—Female proctiger length; **FW**—Forewing width; **GL**—Genal processes length; **HW**—Head width; **MP**—Male proctiger length; **PL**—Paramere length; **SP**—Female subgenital plate length; **TL**—Metatibia length; **UGS**—Urban Green Space; **VL**—Vertex length.

## ﻿Taxonomy

**Psylloidea Latreille, 1807**

### ﻿Aphalaridae Löw, 1879

#### ﻿‡ Ctenarytaina eucalypti

Taxon classificationAnimaliaHemipteraAphalaridae

(Maskell, 1890)

D8EBD340-06E2-52B1-B3B4-A172D589C5CA

##### Material examined.

**Chapinero**: • 1 ♂, 4 ♀; Quebrada La Vieja; 4.6495, –74.0466; 2764 m; 06.iv.2017; J. Duran leg.; *Piperbogotense* (Piperaceae); MPUJ_ENT.

##### Distribution.

Colombia: Boyacá and Bogotá (Pinzón et al. 2002; Rendón-Mera et al. 2017).—Native to Australia, introduced into Africa, the Americas, Asia, Europe, and New Zealand (Makunde et al. 2020).

##### Host plant.

*Eucalyptus* L’Hér. spp. (Myrtaceae) (Makunde et al. 2020).

#### ﻿‡ Ctenarytaina spatulata

Taxon classificationAnimaliaHemipteraAphalaridae

Taylor, 1997

3DDFA55D-86A8-5871-8ED0-BBF7F591D80E

##### Material examined.

**Chapinero**: • 1 ♀; Quebrada La Vieja; 4.6474, –74.0447; 2785 m; 20.vi.2017; J. Duran leg.; *Miconiaelaeoides* (Melastomataceae); MPUJ_ENT. **Santa Fe**: • 4 ♂, 3 ♀; Universidad Distrital; 4.5989, –74.0656; 2701 m; 05.v.2017; J. Duran leg.; *Oreopanaxincisus* (Araliaceae); MPUJ_ENT• 1 ♀; same but 4.5987, –74.0653; 2713 m; *Quercushumboldtii* (Fagaceae); MPUJ_ENT. **Usaquén**: • 1 ♀; Parque Cabañas del Norte; 4.7359, –74.0318; 2575 m; 16.iii.2018; V. Ocampo leg.; *Lafoensiaacuminata* (Lythraceae); MPUJ_ENT • 1 ♀; Parque Contador Norte; 4.715, –74.0302; 2595 m; 02.iv.2018; V. Ocampo leg.; *Liquidambarstyraciflua* (Altingiaceae); MPUJ_ENT.

##### Distribution.

Colombia: Bogotá (Rendón-Mera et al. 2017).—Native to Australia, introduced into the Americas, Europe, and New Zealand (Makunde et al. 2020).

##### Host plant.

*Eucalyptus* L’Hér. spp. (Myrtaceae) (Makunde et al. 2020).

#### ﻿‡ Glycaspis brimblecombei

Taxon classificationAnimaliaHemipteraAphalaridae

Moore, 1964

89043217-29F8-557B-93CD-1FB5B4DFD00D

##### Material examined.

**Chapinero**: • 1 ♀; Parque El Virrey; 4.6736, –74.0548; 2590 m; 28.iii.2017; J. Duran leg.; *Feijoasellowiana* (Myrtaceae); MPUJ_ENT. **Santa Fe**: • 1 ♀; Parque Ilarco; 4.7003, –74.0655; 2569 m; 19.ix.2017; J. Duran leg.; *Clusia* sp. (Clusiaceae); MPUJ_ENT.

##### Distribution.

Colombia: Antioquia, Bogotá, Casanare, Risaralda, and Valle del Cauca (Rodas et al. 2014; Rendón-Mera et al. 2017).—Native to Australia, introduced into Africa, the Americas, Asia, Europe, and New Zealand (Pugh et al. 2017; Makunde et al. 2020).

##### Host plant.

*Corymbia* K.D.Hill and L.A.S.Johnson, and *Eucalyptus* L’Hér. spp. (Myrtaceae) (Makunde et al. 2020).

#### ﻿‡ Syncoptozus mexicanus

Taxon classificationAnimaliaHemipteraAphalaridae

Hodkinson, 1990

7B8EB967-0875-582D-8AFD-FBBB0055C3DC

##### Material examined.

**Antonio Nariño**: • 1 ♂; Parque Ciudad Jardín; 4.5819, –74.0937; 2601 m; 13.iv.2018; V. Ocampo leg.; *Lafoensiaacuminata* (Lythraceae); MPUJ_ENT. **Chapinero**: • 1 ♂; Parque El Virrey; 4.6744, –74.0571; 2580 m; 20.vi.2017; J. Duran leg.; *Fraxinuschinensis* (Oleaceae); MPUJ_ENT • 1 ♂, 1 ♀; same but 4.674, –74.0565; 2581 m; *Ligustrum* sp. (Oleaceae); MPUJ_ENT • 3 ♂, 6 ♀; same but 4.6712, –74.0497; 2583 m; *Magnoliagrandiflora* (Magnoliaceae); MPUJ_ENT • 12 ♂, 16 ♀; same but 4.6753, –74.0581; 2579 m; 28.iii.2017; MPUJ_ENT. **Santa Fe**: • 14 ♂, 17 ♀; Parque Tercer Milenio; 4.5971, –74.0830; 2607 m; 23.iii.2017; J. Duran leg.; *Magnoliagrandiflora* (Magnoliaceae); MPUJ_ENT • 1 ♂, 1 ♀; same but 4.5971, –74.0829; 2606 m; 19.ix.2017; MPUJ_ENT.

##### Distribution.

Colombia: Bogotá (Rendón-Mera et al. 2017), Mexico (Hodkinson 1990).

##### Host plant.

*Magnoliagrandiflora* L. (Magnoliaceae) (unpublished NHMB data from Mexico).

### ﻿Calophyidae Vondráček, 1957

#### ﻿‡ Calophya schini

Taxon classificationAnimaliaHemipteraCalophyidae

Tuthill, 1959

09CC5398-48B7-5A93-B504-247EED7BB84D

##### Material examined.

**Antonio Nariño**: • 1 ♀; Parque Ciudad Jardín; 4.5818, –74.0933; 2601 m; 13.iv.2018; V. Ocampo leg.; *Lafoensiaacuminata* (Lythraceae); MPUJ_ENT • 1 ♀; same but 4.5819, –74.0937; 2601 m; MPUJ_ENT • 58 ♂, 73 ♀; same but 4.5814, –74.0932; 2601 m; *Schinusareira* (Anacardiaceae); MPUJ_ENT • 116 ♂, 104 ♀; same but 4.5816, –74.0931; 2600 m; MPUJ_ENT • 114 ♂, 159 ♀; same but 4.5817, –74.0931; 2602 m; MPUJ_ENT • 1 ♀; same but 4.5821, –74.0914; 2599 m; MPUJ_ENT • 45 ♂, 56 ♀; same but 4.5822, –74.0932; 2597 m; MPUJ_ENT. **Chapinero**: • 1 ♂, 6 ♀; Parque El Chicó; 4.673, –74.0452; 2599 m; 27.iv.2018; V. Ocampo leg.; *Schinusareira* (Anacardiaceae); MPUJ_ENT • 1 ♂, 2 ♀; Parque El Virrey; 4.6754, –74.0581; 2579 m; 25.ix.2017; J. Duran leg.; *Crotoncoriaceus* (Euphorbiaceae); MPUJ_ENT • 6 ♂, 5 ♀; same but 28.iii.2017; MPUJ_ENT • 2 ♂, 2 ♀; same but 4.6739, –74.0557; 2580 m; *Salixhumboldtiana* (Salicaceae); MPUJ_ENT. **Rafael Uribe Uribe**: • 1 ♀; Parque Palermo Sur; 4.5412, –74.1100; 2698 m; 09.iv.2018; V. Ocampo leg.; *Pittosporumundulatum* (Pittosporaceae); MPUJ_ENT • 10 ♂, 16 ♀; same but 4.5417, –74.1097; 2689 m; *Schinusareira* (Anacardiaceae); MPUJ_ENT. **San Cristóbal**: • 1 ♀; Parque La Victoria; 4.5546, –74.0954; 2757 m; 09.iv.2018; V. Ocampo leg.; *Pittosporumundulatum* (Pittosporaceae); MPUJ_ENT • 4 ♂, 1 ♀; same but 4.5548, –74.0955; 2764 m; *Quercushumboldtii* (Fagaceae); MPUJ_ENT • 79 ♂, 92 ♀; same but 4.5546, –74.0953; 2759 m; *Schinusareira* (Anacardiaceae); MPUJ_ENT • 31 ♂, 48 ♀; same but 4.5547, –74.0953; 2760 m; MPUJ_ENT • 3 ♂, 4 ♀; Parque San Cristóbal: 4.5735, –74.0832; 2639 m; 13.iv.2018; V. Ocampo leg.; *Schinusareira* (Anacardiaceae); MPUJ_ENT • 2 ♂, 3 ♀; same but 4.5736, –74.0834; 2638 m; MPUJ_ENT • 60 ♂, 17 ♀; same but 4.5736, –74.0827; 2642 m; MPUJ_ENT • 44 ♂, 37 ♀; Parque Villa de los Alpes; 4.5593, –74.0977; 2692 m; 13.iv.2018; V. Ocampo leg.; *Schinusareira* (Anacardiaceae); MPUJ_ENT. **Santa Fe**: • 4 ♂, 2 ♀; Parque Tercer Milenio; 4.5971, –74.0829; 2606 m; 19.ix.2017; J. Duran leg.; *Magnoliagrandiflora* (Magnoliaceae); MPUJ_ENT • 1 ♂, 7 ♀; same but 4.7011, –74.0655; 2570 m; 23.iii.2017; *Schinusareira* (Anacardiaceae); MPUJ_ENT. **Usaquén**: • 1 ♀; CAI Santa Barbara; 4.693, –74.0311; 2601 m; 06.iv.2018; V. Ocampo leg.; *Quercushumboldtii* (Fagaceae); MPUJ_ENT • 4 ♂, 4 ♀; Parque Belmira; 4.7215, –74.0318; 2576 m; 02.iv.2018; V. Ocampo leg.; *Schinusareira* (Anacardiaceae); MPUJ_ENT • 1 ♂; Parque Cabañas del Norte; 4.7359, –74.0318; 2575 m; 16.iii.2018; V. Ocampo leg.; *Lafoensiaacuminata* (Lythraceae); MPUJ_ENT • 33 ♂, 27 ♀; same but 4.7359, –74.0317; 2574 m; *Schinusareira* (Anacardiaceae); MPUJ_ENT • 102 ♂, 116 ♀; same but 4.7359, –74.0315; 2576 m; MPUJ_ENT • 19 ♂, 18 ♀; same but 4.7359, –74.0316; 2575 m; MPUJ_ENT • 48 ♂, 56 ♀; same but 4.736, –74.0317; 2571 m; MPUJ_ENT • 1 ♀; Parque Contador Norte; 4.7152, –74.0297; 2599 m; 02.iv.2018; V. Ocampo leg.; *Liquidambarstyraciflua* (Altingiaceae); MPUJ_ENT • 19 ♂, 21 ♀; Parque Ginebra-Bella Suiza; 4.7061, –74.0300; 2595 m; 06.iv.2018; V. Ocampo leg.; *Schinusareira* (Anacardiaceae); MPUJ_ENT • 13 ♂, 6 ♀; same but 4.7062, –74.0305; 2596 m; MPUJ_ENT • 55 ♂, 34 ♀; same but 4.7067, –74.0299; 2601 m; MPUJ_ENT • 2 ♂, 1 ♀; Parque La Francia; 4.6896, –74.0470; 2577 m; 05.iii.2018; V. Ocampo leg.; *Schinusareira* (Anacardiaceae); MPUJ_ENT • 1 ♀; same but 4.6902, –74.0464; 2577 m; MPUJ_ENT • 1 ♀; same but 4.6906, –74.0464; 2575 m; MPUJ_ENT • 1 ♂, 1 ♀; same but 4.6908, –74.0466; 2577 m; MPUJ_ENT • 1 ♀; Parque Nueva Autopista; 4.7217, –74.0507; 2579 m; 05.iii.2018; V. Ocampo leg.; *Ficus* sp. (Moraceae); MPUJ_ENT • 1 ♀; Parque Usaquén 2; 4.691, –74.0323; 2586 m; 05.iii.2018; V. Ocampo leg.; *Ficus* sp. (Moraceae); MPUJ_ENT • 1 ♂; same but 4.691, –74.0320; 2591 m; 27.iv.2018; *Liquidambarstyraciflua* (Altingiaceae); MPUJ_ENT • 1 ♀; same but 4.6912, –74.0317; 2571 m; 05.iii.2018; *Pittosporumundulatum* (Pittosporaceae); MPUJ_ENT. **Usme**: • 1 ♂, 1 ♀; Parque Chuniza-Famaco; 4.5018, –74.1086; 2775 m; 09.iv.2018; V. Ocampo leg.; *Quercushumboldtii* (Fagaceae); MPUJ_ENT • 158 ♂, 173 ♀; same but 4.5015, –74.1088; 2686 m; *Schinusareira* (Anacardiaceae); MPUJ_ENT • 28 ♂, 31 ♀; same but 4.5018, –74.1087; 2774 m; MPUJ_ENT • 55 ♂, 44 ♀; same but 4.5031, –74.1100; 2759 m; MPUJ_ENT • 16 ♂, 15 ♀; same but 4.5032, –74.1097; 2761 m; MPUJ_ENT • 20 ♂, 18 ♀; same but 4.5036, –74.1101; 2754 m; MPUJ_ENT • 22 ♂, 29 ♀; Parque Virrey Sur; 4.5009, –74.1125; 2768 m; 23.iv.2018; V. Ocampo leg.; *Schinusareira* (Anacardiaceae); MPUJ_ENT.

##### Distribution.

Colombia: Bogotá (Pinzón and González 2002).—Probably originating from Bolivia or Peru, adventive elsewhere in the Americas, Africa, Europe, and New Zealand (Burckhardt et al. 2018).

##### Host plant.

*Schinusareira* L. (Anacardiaceae) (Burckhardt et al. 2018).

### ﻿Carsidaridae Crawford, 1911

#### ﻿ Synoza cornutiventris

Taxon classificationAnimaliaHemipteraCarsidaridae

Enderlein, 1918

D25F3784-7CFD-5299-AD85-8462FC81DFAD

##### Material examined.

**Antonio Nariño**: • 4 ♂, 5 ♀; Parque Ciudad Jardín; 4.5818, –74.0932; 2601 m; 23.iv.2018; V. Ocampo leg.; *Ficus* sp. (Moraceae); MPUJ_ENT. **Chapinero**: • 2 ♀; Parque El Chicó; 4.6731, –74.0447; 2605 m; 27.iv.2018; V. Ocampo leg.; *Ficus* sp. (Moraceae); MPUJ_ENT • 6 ♂, 2 ♀; same but 4.6732, –74.0445; MPUJ_ENT • 1 ♂; Parque El Virrey; 4.6733, –74.0554; 2591 m; 28.iii.2017; J. Duran leg.; *Delostomaintegrifolium* (Bignoniaceae); MPUJ_ENT. **Rafael Uribe Uribe**: • 2 ♂; Parque Palermo Sur; 4.5423, –74.1102; 2676 m; 09.iv.2018; V. Ocampo leg.; *Ficus* sp. (Moraceae); MPUJ_ENT. **San Cristóbal**: • 8 ♂, 7 ♀; Parque Primero de Mayo; 4.5734, –74.0882; 2625 m; 23.iv.2018; V. Ocampo leg.; *Ficus* sp. (Moraceae); MPUJ_ENT • 28 ♂, 24 ♀; same but 4.5738, –74.0879; 2622 m; 13.iv.2018; MPUJ_ENT • 25 ♂, 14 ♀; same but 4.5745, –74.0881; 2621 m; MPUJ_ENT • 1 ♀; same but 4.5746, –74.0880; 2621 m; *Quercushumboldtii* (Fagaceae); MPUJ_ENT • 1 ♂; Parque San Cristóbal: 4.5728, –74.0848; 2638 m; 13.iv.2018; V. Ocampo leg.; *Lafoensiaacuminata* (Lythraceae); MPUJ_ENT • 1 ♂; same but 4.5728, –74.0838; *Pittosporumundulatum* (Pittosporaceae); MPUJ_ENT • 36 ♂, 24 ♀; Parque Villa de los Alpes; 4.5591, –74.0974; 2698 m; 23.iv.2018; V. Ocampo leg.; *Ficus* sp. (Moraceae); MPUJ_ENT • 11 ♂, 4 ♀; same but 4.5593, –74.0972; 2695 m; MPUJ_ENT • 11 ♂, 16 ♀; same but 4.5595, –74.0978; 2686 m; MPUJ_ENT • 1 ♀; same but 4.5621, –74.0982; 2676 m; 13.iv.2018; MPUJ_ENT • 4 ♂, 6 ♀; same but 4.5624, –74.0983; 2667 m; MPUJ_ENT • 4 ♂, 1 ♀; same but 4.5625, –74.0983; 2665 m; 13.iv.2018; MPUJ_ENT • 2 ♀; same but 4.5628, –74.0982; 2658 m; 23.iv.2018; MPUJ_ENT. **Santa Fe**: • 5 ♂, 1 ♀; Parque La Independencia; 4.6108, –74.0678; 2645 m; 27.iv.2018; V. Ocampo leg.; *Ficus* sp. (Moraceae); MPUJ_ENT • 12 ♂, 11 ♀; same but 4.6114, –74.0682; 2639 m; MPUJ_ENT • 1 ♂; same but 4.6116, –74.0687; 2631 m; MPUJ_ENT • 1 ♂; same but 4.6108, –74.0678; 2644 m; *Liquidambarstyraciflua* (Altingiaceae); MPUJ_ENT • 1 ♂; same but 4.6119, –74.0694; 2583 m; *Quercushumboldtii* (Fagaceae); MPUJ_ENT • 1 ♂; Parque Nacional; 4.6217, –74.0643; 2576 m; 27.iv.2018; V. Ocampo leg.; *Ficus* sp. (Moraceae); MPUJ_ENT • 3 ♀; same but 4.6242, –74.0640; 2624 m; MPUJ_ENT • 1 ♂; Parque Ilarco; 4.7008, –74.0657; 2569 m; 23.iii.2017; J. Duran leg.; *Crotoncoriaceus* (Euphorbiaceae); MPUJ_ENT. **Suba**: • 4 ♂, 5 ♀; Parque Canal Molinos; 4.6981, –74.0634; 2575 m; 23.iii.2017; J. Duran leg.; Ficusamericanasubsp.andicola (Moraceae); MPUJ_ENT. **Usaquén**: • 1 ♂, 1 ♀; Parque Altablanca; 4.7347, –74.0285; 2581 m; 16.iii.2018; V. Ocampo leg.; *Lafoensiaacuminata* (Lythraceae); MPUJ_ENT • 3 ♂, 2 ♀; Parque CAI Lisboa; 4.7085, –74.0290; 2604 m; 06.iv.2018; V. Ocampo leg.; *Pittosporumundulatum* (Pittosporaceae); MPUJ_ENT • 1 ♀; same but 4.7088, –74.0292; 2599 m; MPUJ_ENT • 12 ♂, 5 ♀; Parque Cedro Madeira; 4.7268, –74.0313; 2574 m; 23.iii.2018; V. Ocampo leg.; *Ficus* sp. (Moraceae); MPUJ_ENT • 40 ♂, 24 ♀; Parque Contador Norte; 4.7127, –74.0312; 2594 m; 06.iv.2018; V. Ocampo leg.; *Ficus* sp. (Moraceae); MPUJ_ENT • 13 ♂, 13 ♀; same but 4.7129, –74.0312; 2601 m; MPUJ_ENT • 14 ♂, 13 ♀; same but 4.713, –74.0312; 2598 m; MPUJ_ENT • 1 ♂, 1 ♀; same but 4.7132, –74.0314; 2593 m; *Liquidambarstyraciflua* (Altingiaceae); MPUJ_ENT • 1 ♂; same but 4.7128, –74.0311; 2603 m; *Pittosporumundulatum* (Pittosporaceae); MPUJ_ENT • 1 ♀; Parque Ginebra-Bella Suiza; 4.7067, –74.0298; 2601 m; 06.iv.2018; V. Ocampo leg.; *Schinusareira* (Anacardiaceae); MPUJ_ENT • 2 ♂, 2 ♀; Parque La Francia; 4.6899, –74.0466; 2579 m; 05.iii.2018; V. Ocampo leg.; *Ficus* sp. (Moraceae); MPUJ_ENT • 2 ♀; same but 4.6908, –74.0480; 2580 m; MPUJ_ENT • 2 ♂; same but 4.6905, –74.0475; 2581 m; *Lafoensiaacuminata* (Lythraceae); MPUJ_ENT • 21 ♂, 13 ♀; Parque La Vida; 4.7361, –74.0339; 2585 m; 16.iii.2018; V. Ocampo leg.; *Ficus* sp. (Moraceae); MPUJ_ENT • 39 ♂, 22 ♀; same but 4.7362, –74.0339; 2586 m; MPUJ_ENT • 32 ♂, 32 ♀; same but 4.7365, –74.0341; 2577 m; MPUJ_ENT • 12 ♂, 6 ♀; same but 4.7367, –74.0342; 2579 m; MPUJ_ENT • 17 ♂, 17 ♀; same but 4.7369, –74.0350; 2576 m; MPUJ_ENT • 23 ♂, 10 ♀; same but 4.737, –74.0344; 2573 m; MPUJ_ENT • 1 ♂, 5 ♀; same but 4.7371, –74.0352; 2572 m; *Pittosporumundulatum* (Pittosporaceae); MPUJ_ENT • 1 ♂, 1 ♀; Parque Nueva Autopista; 4.7216, –74.0507; 2571 m; 05.iii.2018; V. Ocampo leg.; *Ficus* sp. (Moraceae); MPUJ_ENT • 1 ♂; same but 4.7217, –74.0507; 2579 m; MPUJ_ENT • 2 ♂, 2 ♀; Parque Usaquén 2; 4.691, –74.0323; 2586 m; 05.iii.2018; V. Ocampo leg.; *Ficus* sp. (Moraceae); MPUJ_ENT • 1 ♂; same but 4.6912, –74.0323; 2587 m; *Pittosporumundulatum* (Pittosporaceae); MPUJ_ENT. **Usme**: • 7 ♂, 12 ♀; Parque Diana Turbay; 4.5478, –74.1015; 2672 m; 23.iv.2018; V. Ocampo leg.; *Ficus* sp. (Moraceae); MPUJ_ENT • 5 ♂, 10 ♀; same but 4.5483, –74.1013; MPUJ_ENT • 14 ♂, 8 ♀; Parque La Andrea; 4.5098, –74.1109; 2741 m; 09.iv.2018; V. Ocampo leg.; *Ficus* sp. (Moraceae); MPUJ_ENT • 39 ♂, 32 ♀; same but 4.5098, –74.1109; 2701 m; MPUJ_ENT • 6 ♂, 7 ♀; Parque Virrey Sur; 4.5009, –74.1115; 2779 m; 23.iv.2018; V. Ocampo leg.; *Ficus* sp. (Moraceae); MPUJ_ENT • 4 ♀; same but 4.5012, –74.1113; 2780 m; MPUJ_ENT • 4 ♂, 1 ♀; same but 4.5013, –74.1114; 2781 m; MPUJ_ENT • 4 ♂, 3 ♀; same but 4.5014, –74.1116; 2778 m; MPUJ_ENT.

##### Distribution.

Colombia: Bogotá, Cundinamarca, Meta (Brown and Hodkinson 1988; Rendón-Mera et al. 2017), Costa Rica, Panama, and Peru (Brown and Hodkinson 1988; Hollis 2000).

##### Host plant.

*Ficushartwegii* Miq. (Moraceae) (Hollis 2000). Several adults were collected in the present study on Ficusamericanasubsp.andicola (Standl.) C.C.Berg. This species has to be confirmed as host. Many adults were collected on unidentified *Ficus* trees. It is possible that these also constitute hosts, but they should be identified to species and examined for psyllid immatures for further conclusions.

### ﻿Liviidae Löw, 1879

#### ﻿* Melanastera

Taxon classificationAnimaliaHemipteraLiviidae

sp.

EE9DD33F-D092-5658-A72B-08263410008C

##### Material examined.

**Chapinero**: • 1 ♀; Quebrada La Vieja; 4.6474, –74.0447; 2785 m; 20.vi.2017; J. Duran leg.; *Miconiaelaeoides* (Melastomataceae); MPUJ_ENT.

##### Distribution.

Colombia: Bogotá.

##### Host plant.

Unknown.

##### Comments.

The single female appears to belong to an undescribed species of *Melanastera*, a predominantly Neotropical genus associated with Melastomataceae, Annonaceae, and other plant families (Burckhardt et al. 2024). This is the first record of the genus from Colombia.

### ﻿Mastigimatidae Bekker-Migdisova, 1973

#### ﻿ Mastigimas colombianus

Taxon classificationAnimaliaHemipteraMastigimatidae

Burckhardt, Queiroz & Drohojowska, 2013

DF505AB8-18C9-5BEE-8706-CE5579EC6068

Fig. 3A–G

##### Material examined.

**Chapinero**: • 17 ♂, 17 ♀; Parque El Virrey; 4.6728, –74.0533; 2581 m; 28.iii.2017; J. Duran leg.; *Cedrelamontana* (Meliaceae); MPUJ_ENT • 2 ♂, 2 ♀; same but NMHB • 2 ♂, 2 ♀; same but slide mounted; NMHB. **Santa Fe**: • 1 ♂; Parque Ilarco; 4.7008, –74.0657; 2569 m; 23.iii.2017; J. Duran leg.; *Crotoncoriaceus* (Euphorbiaceae); MPUJ_ENT. **Suba**: • 9 ♂, 6 ♀; Parque Canal Molinos; 4.6976, –74.0637; 2575 m; 10.vii.2017; J. Duran leg.; *Cedrelamontana* (Meliaceae); MPUJ_ENT • 1 ♂, 1 ♀; same but 03.x.2017; MPUJ_ENT. **Usaquén**: • 1 ♀; Parque Cabañas del Norte; 4.7358, –74.0315; 2578 m; 16.iii.2018; V. Ocampo leg.; *Lafoensiaacuminata* (Lythraceae); MPUJ_ENT.

##### Redescription.

**Figure 3.**
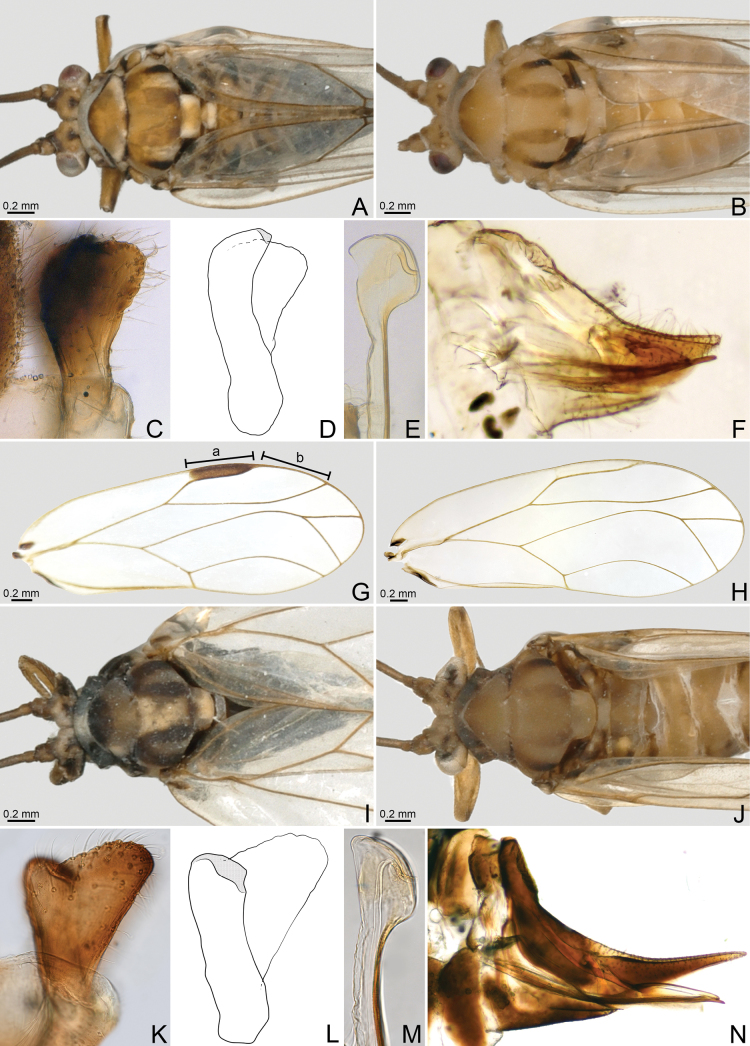
**A–G***Mastigimascolombianus* Burckhardt, Queiroz & Drohojowska, 2013 **H–N***Mastigimaslongicaudatus* Rendón-Mera, Burckhardt & Vargas-Fonseca, sp. nov. **A, I** male, dorsal view **B, J** female, dorsal view **C, K** paramere, outer surface, lateral view **D, L** paramere, inner surface, lateral view **E, M** distal segment of aedeagus, lateral view **F, N** female terminalia, lateral view **G, H** forewing.

***Colouration*.** Male (Fig. 3A) dark yellow with dark brown markings. Vertex with pale brown longitudinal stripe along lateral and anterior margins on either side; discal foveae with dark brown spot; margin of toruli brown. Genal processes and clypeus whitish. Antennal segments 1 and 2 yellow, segment 3 yellow basally, gradually darkening to dark brown apex, segments 4–10 dark brown. Pronotum whitish with lateral sutures brown. Mesopraescutum pale yellow along posterior margins. Mesoscutum with two dark yellow longitudinal stripes on either side, the outer one black posteriorly. Mesoscutellum and metascutellum whitish. Metapostnotum with dark brown spots medially and laterally. Pleura whitish, propleurites black dorsally. Mesosternum brown. Forewing colourless, with black spot at base of C+Sc and basally on anal cell; veins and pterostigma brown. Fore and mid legs with femur dark yellow, tibia and tarsi brown; hind leg with femur dark brown, tibia and tarsi pale yellow. Abdomen brown with yellow spot medially, narrowing to apex; intersegmental membrane straw-coloured. Terminalia dark brown, parameres black, subgenital plate pale yellow dorsally.—Female (Fig. 3B) yellow with only a few black markings. Discal foveae dark yellow. Pronotum with lateral indentations dark yellow. Meso- and metanotum as in male but markings dark yellow, with outermost stripes on mesoscutum black posteriorly. Forewing as in male but pterostigma colourless. Pleura as in male. Fore and mid legs with femora pale yellow, tibiae dark yellow, and tarsi brown, hind leg pale yellow. Terminalia yellow, apex of proctiger black.

***Structure*.** Antenna 4.0–4.1× as long as head width; segment 3 1.3–1.4× as long as segment 4. Forewing (Fig. 3G) 4.5–5.3× as long as head width, and 2.6× as long as wide, pterostigma long and narrow, ratio a/b 0.9–1.0, cell cu_1_ long and flat, length/height ratio 3.3.

***Terminalia*.** Male. Paramere (Fig. 3C, D) bifid, clavate, outer lobe rounded anteriorly and angular posteriorly. Apical dilatation of aedeagus with small blunt apico-ventral hook (Fig. 3E), 1.2× as long as paramere.—Female (Fig. 3F). Terminalia short and cuneate, dorsal outline of proctiger slightly concave; proctiger as long as head width, and 2.4× as long as subgenital plate.

***Measurements*** (in mm). BL 2 ♂ 3.6–5.0 (4.36±0.74), 2 ♀ 5.3–5.4 (5.31±0.08); HW ♂ 0.85, ♀ 0.82; AL ♂ 3.44, ♀ 3.39; FL ♂ 3.8, ♀ 4.36; FW ♂ 1.47, ♀ 1.67; PL ♂ 0.24; DL ♂ 0.28; FP ♀ 0.8; FS ♀ 0.34.

##### Distribution.

Colombia: Bogotá (Burckhardt et al. 2013).

##### Host plant.

Most adults (types and material at hand) were collected on *Cedrelamontana* Turcz. (Meliaceae). *Mastigimas* species develop, as far as known, on *Cedrela*, suggesting that *C.montana* is a host.

##### Comments.

*Mastigimascolombianus* was described from two males and two females collected in Bogotá (Burckhardt et al. 2013). As more material is available from this study, a redescription of the species is provided here. The females in the material at hand fit the original description perfectly but the male paramere is slightly variable with respect to the shape of the outer lobe. As in the two type specimens, the paramere in the material at hand is strongly sclerotised which seems characteristic for the species.

#### ﻿* Mastigimas longicaudatus

Taxon classificationAnimaliaHemipteraMastigimatidae

Rendón-Mera, Burckhardt & Vargas-Fonseca sp. nov.

28962111-4B8A-5089-94C4-BD2D3474B4B6

https://zoobank.org/7AED528B-ED19-4544-96D1-15488A4D5A2F

Fig. 3H–N

##### Type locality.

Colombia, Bogotá: Suba, Parque Canal Molinos, 4.6976389, –74.063694, 2575 m.

##### Type material.

***Holotype***: Colombia • ♂, pinned; Bogotá, Suba, Parque Canal Molinos; 4.6976389, –74.063694; 2575 m; 03.x.2017; J. Duran leg; on *Cedrelamontana* (Meliaceae); MPUJ_ENT0074272. ***Paratypes*: Chapinero**: • 6 ♂, 7 ♀; Parque El Virrey; 4.6728, –74.0533; 2581 m; 28.iii.2017; J. Duran leg.; *Cedrelamontana* (Meliaceae); MPUJ_ENT. **Santa Fe**: • 2 ♀; Parque Tercer Milenio; 4.5974, –74.0835; 2605 m; 23.iii.2017; J. Duran leg.; *Sambucusnigra* (Viburnaceae); MPUJ_ENT. **Suba**: • 8 ♂, 3 ♀; Parque Canal Molinos; 4.6976, –74.0637; 2575 m; 10.vii.2017; J. Duran leg.; *Cedrelamontana* (Meliaceae); MPUJ_ENT • 11 ♂, 13 ♀; same data as for holotype • 1 ♂, 1 ♀; same data as for holotype but NHMB • 1 ♂, 1 ♀; same data as for holotype but slide mounted; NHMB. **Usaquén**: • 1 ♀; Parque Cabañas del Norte; 4.7363, –74.0317; 2575 m; 16.iii.2018; V. Ocampo leg.; *Pittosporumundulatum* (Pittosporaceae); MPUJ_ENT • 1 ♂; Parque Ginebra-Bella Suiza; 4.7061, –74.0304; 2596 m; 06.iv.2018; V. Ocampo leg.; *Liquidambarstyraciflua* (Altingiaceae); MPUJ_ENT • 2 ♂; same but 4.7062, –74.0305; *Schinusareira* (Anacardiaceae); MPUJ_ENT.

##### Diagnosis.

Forewing (Fig. 3H) with pterostigma long and narrow, ratio a/b 1.2 Antennal segment 3 approx. as long as segment 4. Paramere (Fig. 3K, L) bifid, irregularly triangular, strongly widening to apex. Aedeagal head lacking apico-ventral hook. Female terminalia (Fig. 3N) elongate, falcate; proctiger 1.0–1.2× as long as head width.

##### Description.

***Colouration*.** Male (Fig. 3I) dark brown. Head pale yellow; vertex with pale brown longitudinal stripe along lateral and anterior margins on either side; discal foveae with dark brown spot, sometimes much expanded; margin of toruli brown. Genal processes and clypeus whitish. Antennae yellowish brown. Pronotum whitish with lateral quarter dark brown. Mesopraescutum with dark brown polygon-shaped spot anteriorly. Mesoscutum with two dark brown longitudinal stripes on either side. Mesoscutellum and metascutellum whitish. Metapostnotum dark brown. Pleura whitish, with dark brown markings dorsally. Mesosternum dark brown. Forewing colourless, with brown spot at base of C+Sc and base of anal cell; veins yellow; pterostigma dark brown or yellowish brown. Fore and mid legs brown and yellowish brown, metafemur and base of metatibia dark brown, rest of hind leg pale yellow. Abdomen dark brown; intersegmental membrane straw-coloured. Terminalia dark brown, parameres sometimes dark yellow.—Female (Fig. 3J) yellow. Discal foveae dark brown; margin of toruli brown. Pronotum with lateral indentations brown. Tergum as in male but markings dark yellow on mesopraescutum and brownish on mesoscutum, with outermost stripe dark brown posteriorly. Forewing as in male but pterostigma colourless. Pleura and legs as in male. Abdominal sclerites usually brown laterally. Terminalia yellow, brown apically, proctiger sometimes completely brown.

***Structure*.** Conforms to the generic description of Brown and Hodkinson (1988). Antenna 4.3–4.5× as long as head width; segment 3 1.1–1.3× as long as segment 4. Forewing (Fig. 3H) 4.8–5.2× as long as head width, and 2.6–2.7× as long as wide, pterostigma long and narrow, ratio a/b 1.1–1.2, cell cu_1_ long and flat, length/height ratio 3.5–3.8.

***Terminalia*.** Paramere (Fig. 3K, L), bifid, in lateral view irregularly triangular, strongly widening to apex. Apical dilatation of aedeagus lenticular (Fig. 3M), 1.5× as long as paramere.—Female terminalia (Fig. 3N) elongate, falcate; proctiger 1.5× as long as head width, and 1.9× as long as subgenital plate.

***Measurements*** (in mm). BL ♂ 4.8, 2 ♀ 5.8–5.9 (5.85±0.1); HW ♂ 0.88, ♀ 0.92; AL ♂ 3.95, ♀ 3.93; FL ♂ 4.23, ♀ 4.78; FW ♂ 1.57, ♀ 1.85; PL ♂ 0.24; DL ♂ 0.36; FP ♀ 1.26; FS ♀ 0.65.

##### Etymology.

From Latin *longus* = long, and *caudatus* = bearing a tail, referring to the long female terminalia. Adjective.

##### Distribution.

Colombia: Bogotá.

##### Host plant.

Most of the examined adults were collected on *Cedrelamontana* Turcz. (Meliaceae). *Mastigimas* species develop, as far as known, on *Cedrela*, suggesting that *C.montana* is a host.

##### Comments.

*Mastigimaslongicaudatus* Rendón-Mera, Burckhardt & Vargas-Fonseca, sp. nov. resembles *M.anjosi*Burckhardt et al., 2011 (known from Brazil, Trinidad, and Venezuela) in the irregularly triangular paramere and the elongate, falcate female terminalia; it differs in the antennal segment 3 approx. as long as segment 4 (instead of twice as long), and the aedeagal head lacking an apico-ventral hook (Burckhardt et al. 2011, 2013). The falcate female terminalia are shared also with *M.drepanodis* Burckhardt, Queiroz & Drohojowska, 2013 (Brazil) which differs in the slenderer paramere (Burckhardt et al. 2013). In the key by Burckhardt et al. (2013), the new species keys out with *M.colombianus* from which it differs in details of the male and female terminalia.

### ﻿Psyllidae Latreille, 1807

#### ﻿‡ Acizzia acaciaebaileyanae

Taxon classificationAnimaliaHemipteraPsyllidae

(Froggatt, 1901)

6688CCC2-7EDF-5FF0-A0E2-151AA34573F5

##### Material examined.

**Suba**: • 1 ♂, 3 ♀; Cerro La Conejera; 4.7705, –74.0656; 2620 m; 28.iii.2017; J. Duran leg.; *Acaciadealbata* (Fabaceae); MPUJ_ENT • 1 ♀; Parque Ilarco; 4.701, –74.0663; 2567 m; 23.iii.2017; J. Duran leg.; *Bocconiafrutescens* (Papaveraceae); MPUJ_ENT • 1 ♂; same but 10.vii.2017; MPUJ_ENT. **Usaquén**: • 1 ♂; Parque Belmira; 4.7215, –74.0318; 2576 m; 02.iv.2018; V. Ocampo leg.; *Schinusareira* (Anacardiaceae); MPUJ_ENT • 5 ♂; Parque Cabañas del Norte; 4.7358, –74.0315; 2578 m; 16.iii.2018; V. Ocampo leg.; *Lafoensiaacuminata* (Lythraceae); MPUJ_ENT • 1 ♂, 1 ♀; Parque Contador Norte; 4.7158, –74.0322; 2581 m; 06.iv.2018; V. Ocampo leg.; *Lafoensiaacuminata* (Lythraceae); MPUJ_ENT • 1 ♂; same but 4.715, –74.0301; 2597 m; 02.iv.2018; *Liquidambarstyraciflua* (Altingiaceae); MPUJ_ENT • 1 ♀; same but 4.7151, –74.0299; 2600 m; MPUJ_ENT.

##### Distribution.

Colombia: Bogotá (Rendón-Mera et al. 2017).—Native to Australia, adventive in Africa, North America, Asia, Europe, and New Zealand (Ouvrard 2023).

##### Host plant.

*Acacia* Mill. and *Samanea* (Benth.) Merr. spp. (Fabaceae) (Ouvrard 2023).

#### ﻿‡ Acizzia uncatoides

Taxon classificationAnimaliaHemipteraPsyllidae

(Ferris & Klyver, 1932)

94BC505B-D1A6-5B09-9E3C-AE8531FE53AA

##### Material examined.

**Chapinero**: • 9 ♂, 10 ♀; Parque El Virrey; 4.6713, –74.0504; 2591 m; 28.iii.2017; J. Duran leg.; *Acaciamelanoxylon* (Fabaceae); MPUJ_ENT • 18 ♂, 10 ♀; same but 4.6753, –74.0581; 2579 m; *Magnoliagrandiflora* (Magnoliaceae); MPUJ_ENT • 1 ♀; same but 4.6738, –74.0563; 2581 m; *Pittosporumundulatum* (Pittosporaceae); MPUJ_ENT • 1 ♂; Quebrada La Vieja; 4.6495, –74.0466; 2764 m; 06.iv.2017; J. Duran leg.; *Piperbogotense* (Piperaceae); MPUJ_ENT. **Rafael Uribe Uribe**: • 1 ♂; Parque Palermo Sur; 4.542, –74.1089; 2692 m; 09.iv.2018; V. Ocampo leg.; *Pittosporumundulatum* (Pittosporaceae); MPUJ_ENT. **Santa Fe**: • 9 ♂, 6 ♀; Universidad Distrital; 4.5991, –74.0656; 2695 m; 19.ix.2017; J. Duran leg.; *Acaciadecurrens* (Fabaceae); MPUJ_ENT • 1 ♂, 1 ♀; same but 4.5986, –74.0656; 2702 m; 05.v.2017; *Acaciamelanoxylon* (Fabaceae); MPUJ_ENT • 71 ♂, 52 ♀; same but 4.5987, –74.0667; 2667 m; 19.ix.2017; MPUJ_ENT • 1 ♂, 1 ♀; same but 4.5983, –74.0654; 2712 m; 05.v.2017; *Crotoncoriaceus* (Euphorbiaceae); MPUJ_ENT • 10 ♂, 14 ♀; same but 4.5985, –74.0655; 2704 m; *Lyciantheslycioides* (Solanaceae); MPUJ_ENT • 1 ♂; same but 4.5989, –74.0656; 2701 m; *Oreopanaxincisus* (Araliaceae); MPUJ_ENT • 1 ♂; same but 4.5987, –74.0653; 2713 m; *Quercushumboldtii* (Fagaceae); MPUJ_ENT. **Suba**: • 2 ♀; Cerro La Conejera; 4.7718, –74.0648; 2622 m; 10.vii.2017; J. Duran leg.; *Acaciamelanoxylon* (Fabaceae); MPUJ_ENT • 1 ♂; same but 4.7695, –74.0527; 2674 m; 03.x.2017; *Quercushumboldtii* (Fagaceae); MPUJ_ENT. **Usaquén**: • 1 ♂; Parque CAI Lisboa; 4.7088, –74.0292; 2599 m; 06.iv.2018; V. Ocampo leg.; *Pittosporumundulatum* (Pittosporaceae); MPUJ_ENT • 1 ♂; same but 4.7094, –74.0291; 2590 m; MPUJ_ENT.

##### Distribution.

Colombia: Bogotá, Cundinamarca, Huila (Rendón-Mera et al. 2017).—Native to Australia, adventive in Africa, the Americas, Asia, Europe, North Africa, and New Zealand (Ouvrard 2023).

##### Host plant.

*Acacia* Mill. and *Albizia* A. ex Benth. (Fabaceae) (Halbert and Burckhardt 2020); in this survey several adults were collected on *Acaciadecurrens* (J.C.Wendl.) Willd. and *A.melanoxylon* R.Br. While the latter is confirmed in the literature as host, the former is not. Further studies will be necessary to find out whether *A.decurrens* serves as host to *A.uncatoides*.

#### ﻿ Platycorypha

Taxon classificationAnimaliaHemipteraPsyllidae

sp.

9A01B3A7-D758-537B-89A0-05202973E3D3

##### Material examined.

**Santa Fe**: • 1 ♀; Parque Ilarco; 4.7008, –74.0657; 2569 m; 23.iii.2017; J. Duran leg.; *Crotoncoriaceus* (Euphorbiaceae); MPUJ_ENT.

##### Distribution.

Colombia: Bogotá, Magdalena (Rendón-Mera et al. 2017).

##### Host plant.

Unknown. The single female at hand was collected on *Croton*, an unlikely host as all *Platycorypha* species, for which hosts are known, develop on Fabaceae (Burckhardt and Queiroz 2020).

##### Comments.

The single female at hand resembles specimens reported as *Platycoryphaerythrinae* (Lizer) from Panama (Brown and Hodkinson 1988) and Peru (Burckhardt 1987). These specimens are probably not conspecific with *P.erythrinae* from Argentina, Brazil, Paraguay, and Uruguay, but represent an undescribed species. The specimens from Colombia, Panama and Peru differ from the latter in the presence of distinct brown dots on the radular areas of the forewing and the small hook on the apex of the female proctiger. More material is required for solving this issue.

#### ﻿ Tuthillia latipennis

Taxon classificationAnimaliaHemipteraPsyllidae

Hodkinson, Brown & Burckhardt, 1986

3B5C6D03-47BF-5181-B8D7-E8600F7370CD

##### Material examined.

**Suba**: • 1 ♀; Cerro La Conejera; 4.7718, –74.0651; 2631 m; 03.x.2017; J. Duran leg.; *Myrcianthesleucoxyla* (Myrtaceae); MPUJ_ENT. **Engativá**: • 1 ♂; Jardín Botánico de Bogotá; 4.6666, –74.0993; 2553 m; 16.x.2019; S. Vargas leg.; *Myrcianthes* sp. (Myrtaceae); MPUJ_ENT.

##### Distribution.

Colombia: Bogotá (Rendón-Mera et al. 2017), Costa Rica, Panama (Brown and Hodkinson 1988; Hollis 2000).

##### Host plant.

*Myrcianthesfragrans* (Sw.) McVaugh (Myrtaceae) (Hollis 2000). If *Myrcianthesleucoxyla* (Ortega) McVaugh, on which one female was collected, also constitutes a host, needs further observations.

### ﻿Triozidae Löw, 1879

#### ﻿ Calinda gibbosa

Taxon classificationAnimaliaHemipteraTriozidae

(Tuthill, 1959)

60355E31-B6A1-5065-952B-DAB7F87E5466

##### Material examined.

**Santa Fe**: • 1 ♂, 2 ♀; Universidad Distrital; 4.5995, –74.0664; 2673 m; 19.ix.2017; J. Duran leg.; *Baccharislatifolia* (Asteraceae); MPUJ_ENT • 1 ♀; same but 4.5997, –74.0653; 2692 m; *Quercushumboldtii* (Fagaceae); MPUJ_ENT. **Suba**: • 1 ♀; Cerro La Conejera; 4.7702, –74.0664; 2634 m; 10.vii.2017; J. Duran leg.; *Baccharis* sp. (Asteraceae); MPUJ_ENT.

##### Distribution.

Colombia: Antioquia, Bogotá, Boyacá, Cundinamarca, Nariño (Olivares and Burckhardt 1997; Rendón-Mera et al. 2017), Cuba, Ecuador, Peru, Venezuela (Olivares and Burckhardt 1997).

##### Host plant.

*Baccharislatifolia* Pers. (Asteraceae) (Olivares and Burckhardt 1997).

#### ﻿* Calinda trinervis

Taxon classificationAnimaliaHemipteraAsteraceae

Olivares & Burckhardt, 1997

E91217EC-CE71-51F6-B30E-BCE42D784B62

##### Material examined.

**Santa Fe**: • 1 ♀; Universidad Distrital; 4.5995, –74.0664; 2673 m; 19.ix.2017; J. Duran leg.; *Baccharislatifolia* (Asteraceae); MPUJ_ENT.

##### Distribution.

Colombia: Bogotá, Costa Rica, Panama (Olivares and Burckhardt 1997).

##### Host plant.

Unknown. Adults from Colombia were collected on *Baccharislatifolia* Pers. and adults from Costa Rica on *B.trinervis* Pers. (Asteraceae). Both should be checked to determine whether they are hosts.

##### Comments.

*Calindatrinervis* is reported here for the first time from Colombia.

#### ﻿ Calinda

Taxon classificationAnimaliaHemipteraAsteraceae

sp.

A2EDB7F9-024E-5C33-8AF1-BE3451909F4B

##### Material examined.

**Santa Fe**: • 1 ♀; Parque Ilarco; 4.7008, –74.0657; 2569 m; 23.iii.2017; J. Duran leg.; *Crotoncoriaceus* (Euphorbiaceae); MPUJ_ENT.

##### Distribution.

Colombia: Bogotá.

##### Host plant.

Unknown.

##### Comments.

The single female at hand represents probably an undescribed species. It shares the following characters with *Calindaalbonigra* Olivares & Burckhardt, 1997 and *C.gladiformis* Olivares & Burckhardt, 1997: antenna shorter than 1.2 mm; forewing lacking surface spinules in distal 1/2; apical projection of proctiger well delimited from base, not inflated, straight, pointed apically, with well-defined teeth along dorsal margin; subgenital plate long; valvula dorsalis long; ventral saw of valvula ventralis not well delimited at base. From the former it differs in the relatively longer processes on the proctiger and subgenital plate. From the latter it differs in the relatively shorter apical process of the proctiger and the presence of a small ventral hump in the basal 1/3 of the subgenital plate.

#### ﻿* Leuronota albilinea

Taxon classificationAnimaliaHemipteraAsteraceae

Rendón-Mera, Burckhardt & Vargas-Fonseca sp. nov.

58A1AB79-83A8-5420-B7BE-2EF628E2CCC5

https://zoobank.org/B778B877-DF95-4CAD-9A27-D325DE459AF0

Fig. 4

##### Type locality.

Colombia, Bogotá: Santa Fe, Parque Tercer Milenio, 4.70025, –74.0654667, 2569 m.

##### Type material.

***Holotype***: Colombia • ♂, pinned; Bogotá, Santa Fe, Parque Tercer Milenio; 4.70025, –74.0654667; 2569 m; 19.ix.2017; J. Duran leg.; on *Clusia* sp. (Clusiaceae); MPUJ_ENT0074271. ***Paratypes*: Santa Fe**: • 28 ♂, 23 ♀; same data as for holotype but 4.7003, –74.0655; MPUJ_ENT • 1 ♂, 1 ♀; same data as for preceding but NHMB • 1 ♂, 1 ♀; same data as for preceding but slide mounted; NHMB • 4 ♂, 4 ♀; same data as for preceding but in ethanol 70%; NHMB • 19 ♂, 11 ♀; same data as for preceding but 27.vi.2017; MPUJ_ENT • 1 ♂; same data as for preceding but 4.5989, –74.0814; 2607 m; *Prunusserotina* (Rosaceae); MPUJ_ENT.

##### Diagnosis.

**Figure 4.**
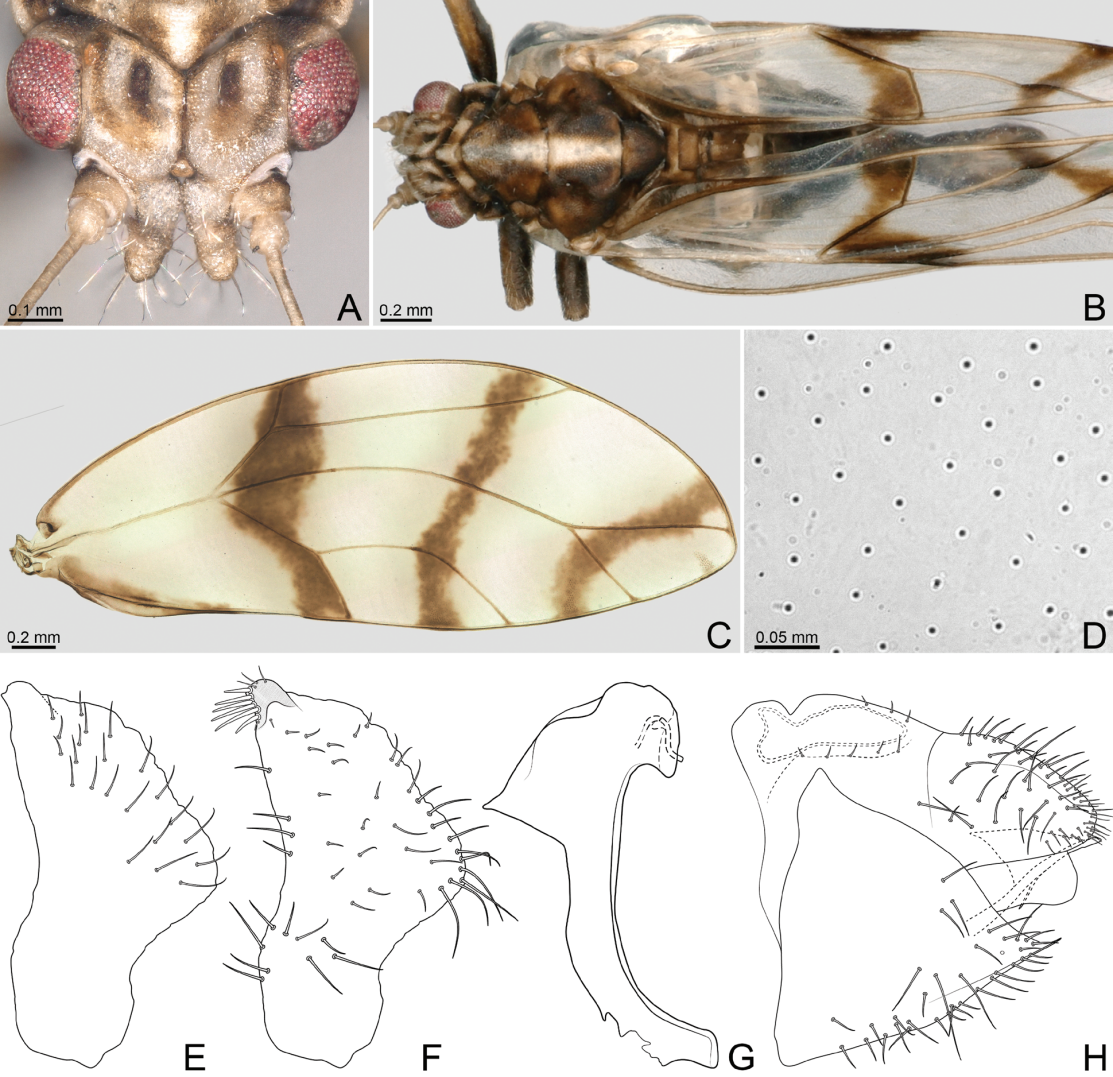
*Leuronotaalbilinea* Rendón-Mera, Burckhardt & Vargas-Fonseca, sp. nov. **A** head, dorsal view **B** habitus, dorsal view **C** forewing **D** surface spinules **E–G** male terminalia, lateral view **E** paramere, outer surface **F** paramere, inner surface **G** distal segment of aedeagus **H** female terminalia, lateral view.

Mesonotum with white longitudinal stripe (Fig. 4B). Forewing (Fig. 4C) with three brown transverse bands as follows: one along vein R_1_, base of cells r_2_ and m_2_, vein Cu_1_ and apex of cell cu_2_ adjacent to vein Cu_1b_, one from subapex of cell r_1_, through approx. middle of r_2_ and m_2_, to radular spinules of cu_1_, and one from subapex of r_2_, through base of m_1_ to radular spinules of m_2_; clavus brown along A_1_ distal to apex of Cu_2_. Paramere (Fig. 4E, F) with apical process short and posterior margin with apical 1/2 sinuous. Female proctiger (Fig. 4H) with apical portion relatively slender.

##### Description.

***Colouration*.** Head, pronotum and pleura white, rest of notum and abdomen dark brown (Fig. 4A, B). Vertex with dark brown longitudinal stripes adjacent to eyes, curving inwards distal to torulus; anterior margin usually brownish; discal foveae dark brown; margin of toruli brown. Genal process sometimes slightly darker apically. Antennal segments 1–8 pale-yellow, 9–10 black. Clypeus white, slightly brown posteriorly. Pronotum with two brown longitudinal stripes medially; sublateral and lateral indentations dark brown. Mesopraescutum and mesoscutum with white longitudinal stripe medially. Forewing membrane (Fig. 4C) colourless, with three brown transverse bands as follows: one along vein R_1_, base of cells r_2_ and m_2_, vein Cu_1_ and apex of cell cu_2_ adjacent to vein Cu_1b_, one from near apex of cell r_1_, through approx. middle of r_2_ and m_2_, to radular spinules of cu_1_, and one from near apex of r_2_, through base of m_1_ to radular spinules of m_2_; clavus brown along A_1_ distal to apex of Cu_2_; veins yellow, brown within the colour pattern; radular spinules brown. Fore, mid legs and metafemur brown, rest of hind leg yellow with apicotarsus brown. Abdominal basal sternites white or yellow medially; intersegmental membrane straw-coloured. Male terminalia dark brown. Female terminalia brown dorsally and ventrally, yellow apically.

***Structure*.** Genal processes (Fig. 4A) 1.1–1.3× as long as vertex along midline, subcylindrical, slightly narrowing apically, sometimes slightly curved outwards, divergent; apex rounded. Antenna 3.1–3.6× as long as head width; longest terminal seta 3.5–4.0× as long as short seta, and 0.6–0.8× as long as segment 10. Labium with apical segment 0.3–0.4× as long as medial segment. Forewing (Fig. 4C) 5.2–5.6× as long as head width, and 2.5–2.6× as long as wide, obovate with angular apex; vein C+Sc evenly curved; vein Rs straight; vein M 2.1–2.3× as long as M_1+2_, bifurcating after imaginary line between apices of veins Rs and Cu_1a_; vein M_1+2_ reaching wing margin approximately at imaginary line through trifurcation of vein R+M+Cu and bifurcation of vein M; vein Cu 1.3–1.4× as long as R, and 1.6–1.8× as long as Cu_1b_; cell r_1_ approx. as wide as the narrowest section of r_2_. Surface spinules widely spaced (Fig. 4D), covering m_1_, cu_1_, and cu_2_, and colour pattern on r_2_ and m_2_. Radular spinules forming triangular fields. Metafemur with six or seven apical bristles; metatibia 1.2–1.4× as long as head width.

***Terminalia*.** Male proctiger, in lateral view, subconical; apex constricted at anterior margin; anus large, occupying most part of apex, obliquely blunt. Paramere, in lateral view (Fig. 4E, F), 0.9× as long as proctiger; apical process short; bearing posterior lobe; anterior margin sinuous, concave submedially; posterior margin strongly irregular, concave in basal 1/3, strongly convex in median 1/3, sinuous in apical 1/3; outer surface (Fig. 4E) covered in medium long setae along posterior apical 1/2; inner surface (Fig. 4F) covered in short setae medially, long setae along anterior and posterior margins, and thick bristles anteriorly on apical tooth. Apical dilatation of aedeagus (Fig. 4G) with ventral extension beak-like, short; apically slightly convex, with small subapical hump; sclerotised end tube of ductus ejaculatorius short, weakly sinuate.—Female proctiger (Fig. 4H), in lateral view, 0.9× as long as head width; apical portion ~ 1/2 proctiger length; dorsal outline weakly incised at transverse groove, apical portion relatively slender, apex blunt; covered in long setae laterally, medium long setae dorsally, and short setae apically. Circumanal ring 0.4× as long as proctiger. Subgenital plate (Fig. 4H), in lateral view, 0.8× as long as proctiger; ventral outline straight in basal 1/2, weakly angular in the middle, straight in apical 1/2; sparsely covered in long setae, mostly ventrally and apically.

***Measurements*** (in mm). BL 2 ♂ 3.7–4.1 (3.8±0.31), 2 ♀ 4.4–4.7 (4.79±0.37); HW ♂ 0.63, ♀ 0.67; VL ♂ 0.19, ♀ 0.18; GL ♂ 0.21, ♀ 0.23; AL ♂ 2.2, ♀ 1.78; LAB2 ♂ 0.22, ♀ 0.27; LAB3 ♂ 0.09, ♀ 0.09; FL ♂ 3.36, ♀ 3.46; TL ♂ 0.85, ♀ 0.78; MP 0.29; PL 0.26; FP 0.57; CRL 0.21; AP 0.25; SP 0.47.

##### Etymology.

From Latin *albus* = white, and *linea* = line, referring to the contrasting longitudinal white stripe on the mesonotum. Noun in the ablative case.

##### Distribution.

Colombia: Bogotá.

##### Host plant.

Unknown. Many adults were collected on *Clusia* sp. (Clusiaceae) in the same area suggesting it is a host rather than just a casual plant. Further studies are necessary to to check this assumption.

##### Comments.

*Leuronotaalbilinea* Rendón-Mera, Burckhardt & Vargas-Fonseca, sp. nov. resembles *L.inusitata* (Tuthill, 1944) (known from Costa Rica, Mexico, and Panama) in the brown body colour with white head and pleura, and white longitudinal stripe on mesonotum. It differs in the forewing pattern, the obovate forewing (vs ovate), the paramere with short (vs long) apical process and sinuous (vs concave) apical 1/2 of posterior margin, the aedeagal head with a weakly sinuous apical margin (vs evenly convex), and the female proctiger with a relatively slender apical portion (vs massive). In the key of Brown and Hodkinson (1988), *L.albilinea* Rendón-Mera, Burckhardt & Vargas-Fonseca, sp. nov. keys out with *L.inusitata*. In the key of Burckhardt (1988), the species keys out in couplet 4 with *L.digitulata* Burckhardt, 1988 (Paraguay) and *L.fagarae* Burckhardt, 1988 (Brazil, Ecuador, Mexico, Paraguay, USA), from which it differs in the forewing pattern with three brown transverse bands (vs restricted to anal margin or completely or almost completely covering the entire membrane).

#### ﻿* Triozidae

Taxon classificationAnimaliaHemipteraTriozidae

gen. sp. 1

B6435409-A562-5887-B10B-BEFF81457DC4

##### Material examined.

**Engativá**: • 1 ♂; Jardín Botánico de Bogotá; 4.6666, –74.0993; 2553 m; 16.x.2019; S. Vargas leg.; *Myrcianthes* sp. (Myrtaceae); MPUJ_ENT. **Suba**: • 1 ♂; Cerro La Conejera; 4.7695, –74.0527; 2674 m; 03.x.2017; J. Duran leg.; *Quercushumboldtii* (Fagaceae); MPUJ_ENT.

##### Distribution.

Colombia: Bogotá.

##### Host plant.

Unknown.

##### Comments.

The two males at hand probably represent an undescribed species. More material is required for a proper identification.

#### ﻿* Triozidae

Taxon classificationAnimaliaHemipteraTriozidae

gen. sp. 2

A27DB3F5-A342-5A9A-AF17-26588122FA5B

##### Material examined.

**Rafael Uribe Uribe**: • 1 ♀; Parque Palermo Sur; 4.5413, –74.1097; 2692 m; 09.iv.2018; V. Ocampo leg.; *Pittosporumundulatum* (Pittosporaceae); MPUJ_ENT.

##### Distribution.

Colombia: Bogotá.

##### Host plant.

Unknown.

##### Comments.

The single female at hand fits in the *Triozapsyllihabitus* species group of Brown and Hodkinson (1988). More material is required for a species identification.

#### ﻿* Triozidae

Taxon classificationAnimaliaHemipteraTriozidae

gen. sp. 3

FC56B880-2559-587D-816A-8B5B6D08F249

##### Material examined.

**Santa Fe**: • 1 ♂; Universidad Distrital; 4.5987, –74.0653; 2713 m; 27.vi.2017; J. Duran leg.; *Quercushumboldtii* (Fagaceae); MPUJ_ENT. **Usaquén**: • 1 ♀; Parque Ginebra-Bella Suiza; 4.706, –74.0302; 2591 m; 06.iv.2018; V. Ocampo leg.; *Ficus* sp. (Moraceae); MPUJ_ENT • 1 ♀; Parque La Vida; 4.7362, –74.0339; 2586 m; 16.iii.2018; V. Ocampo leg.; *Ficus* sp. (Moraceae); MPUJ_ENT.

##### Distribution.

Colombia: Bogotá.

##### Host plant.

Unknown.

##### Comments.

The three specimens share the conspicuous dark longitudinal stripe on the forewing with species of *Triozoida* Crawford, 1911, a feature also found in other unrelated species of Triozidae (unpublished NHMB data). More material is required for a species identification.

## ﻿Discussion and conclusions

During the survey of the arthropod fauna of 33 UGS in Bogotá between 2017 and 2019, 3,825 adult specimens of 21 psyllid species of seven families were found, seven species of which could be identified only to genus. Psyllids were found in all UGS ranging from 1–8 species per UGS. The UGS with the highest number (8 spp.) is Parque Ilarco, followed by Parque El Virrey (7 spp.), Parque Cabañas del Norte (5 spp.) and Universidad Distrital (Pueblo Viejo) (5 spp.) (Table 1). Parque El Virrey serves as a “contemplative” park while the other three UGS are designed for different purposes, primarily recreational use, and two of them, viz. Parque Ilarco and Parque Cabañas del Norte, are small parks with an area of less than 1 hectare each (Alcaldía Mayor de Bogotá 2021, 2022). At first sight this may be surprising, and one would expect that larger UGS specifically designed for conservation purposes would support the largest number of psyllid species. As psyllids are host specific, the presence of the host is the most important factor allowing the occurrence of psyllid species at a particular place. Local psyllid diversity usually reflects local host diversity.

The number of 21 species found during the survey is high in comparison to the number of taxa previously reported from Colombia: 34 identified species plus ten species identified only to genus (Pinzón et al. 2002; Rendón-Mera et al. 2017). This high percentage is, however, an artefact of the poor knowledge of the psyllid fauna of Colombia. From Brazil, whose psyllid diversity is slightly better known than that of Colombia, 163 species have been recorded (Burckhardt and Queiroz 2023). However, the actual number of species is likely to be in excess of 1000 (Burckhardt and Queiroz 2020). Comparing the number of plant species of the two countries with 44,000 species in Brazil and 37,000 species in Colombia (Flora e Funga do Brasil 2023; SiB Colombia 2023), it is reasonable to expect several hundreds of psyllid species in Colombia. The presence of previously undescribed species and the high percentage (38%) of species identified only to genus is a further indication of the hazy state of taxonomic knowledge.

Most specimens (3,800) were taken on plants which we consider hosts (vs 184 on non-hosts) (Table 2). Among the seven species with more than 20 collected specimens, less than 10% of the specimens were collected on non-hosts for four of them, while two species had between 10 and 15% of specimens on non-hosts. In only one species, *Acizziauncatoides*, almost 40% of specimens were collected on non-hosts, reflecting the high mobility of this invasive species. Of these seven species, two, viz. *Calophyaschini* and *Syncoptozusmexicanus*, are known to be monophagous, while the others are oligophagous. The suspected hosts of *Leuronotaalbilinea* Rendón-Mera, Burckhardt & Vargas-Fonseca, sp. nov. (*Clusia* sp.), *Mastigimascolombianus* (*Cedrelamontana*), *M.longicaudatus* Rendón-Mera, Burckhardt & Vargas-Fonseca, sp. nov. (*Cedrelamontana*) and *Synozacornutiventris* (Ficusamericanasubsp.andicola, *Ficus* sp.) are native, probably including those not identified to species.

A third of the psyllid species and more than 70% of the specimens found during the survey are exotic: the Australian *Acizziaacaciaebaileyanae*, *A.uncatoides*, *Ctenarytainaeucalypti*, *C.spatulata* and *Glycaspisbrimblecombei*, the North American *Syncoptozusmexicanus*, and the Peruvian *Calophyaschini*. The high abundance of these species is promoted by urban landscaping practices using exotic tree species (Molina-Prieto and Acosta-Hernández 2018; Bernal et al. 2022; Molina 2022), such as *Acaciadecurrens*, *A.melanoxylon*, *Schinusareira*, and *Magnoliagrandiflora*. Incidently, species like *A.decurrens*, *A.melanoxylon*, *Eucalyptusglobulus*, and *S.areira* were among the earliest species used for urban arborisation in Bogotá (Molina-Prieto and Acosta-Hernández 2018). *Schinusareira*, the host of *C.schini*, the most abundant psyllid species of the survey (63% of all specimens), constitutes one of the most characteristic trees of Bogotá (Jardín Botánico de Bogotá 2022). Native to Bolivia, northern Chile, and Peru (Bernal et al. 2016; POWO 2023), *S.areira* was introduced into Bogotá around 1850 (Molina-Prieto and Acosta-Hernández 2018) and now numbers approximately 24,000 trees (Jardín Botánico de Bogotá 2023). Immatures of *C.schini* induce pit-galls on the leaflets of their host (Pinzón and González 2002; Rendón-Mera et al. 2017), and it is not uncommon to find the heavily galled foliage of *S.areira* throughout the city (pers. observation of the authors).

There are twice as many native as exotic psyllid species (66%) but only four of these (*Leuronotaalbilinea* Rendón-Mera, Burckhardt & Vargas-Fonseca, sp. nov., *Mastigimascolombianus*, *M.longicaudatus* Rendón-Mera, Burckhardt & Vargas-Fonseca, sp. nov., and *Synozacornutiventris*) are represented by more than five individuals. Of the other ten species, three are identified to species and the other seven may be undescribed, but more material is needed to confirm this.

The psyllid data from our arthropod survey show that the UGS in Bogotá support a diverse psyllid fauna. The dominance of exotic tree species (Jardín Botánico de Bogotá 2023), however, promotes adventive, potentially invasive psyllids at the expense of the native fauna. For conservation of the native insect fauna, the use of native trees and shrubs should be considered a priority when new UGS are planned.

## Supplementary Material

XML Treatment for Ctenarytaina eucalypti

XML Treatment for Ctenarytaina spatulata

XML Treatment for Glycaspis brimblecombei

XML Treatment for Syncoptozus mexicanus

XML Treatment for Calophya schini

XML Treatment for Synoza cornutiventris

XML Treatment for Melanastera

XML Treatment for Mastigimas colombianus

XML Treatment for Mastigimas longicaudatus

XML Treatment for Acizzia acaciaebaileyanae

XML Treatment for Acizzia uncatoides

XML Treatment for Platycorypha

XML Treatment for Tuthillia latipennis

XML Treatment for Calinda gibbosa

XML Treatment for Calinda trinervis

XML Treatment for Calinda

XML Treatment for Leuronota albilinea

XML Treatment for Triozidae

XML Treatment for Triozidae

XML Treatment for Triozidae

## ﻿Additional information

### Author ORCIDs

Diana Isabel Rendón-Mera https://orcid.org/0000-0003-2553-9486

Daniel Burckhardt https://orcid.org/0000-0001-8368-5268

Sergio Andrés Vargas-Fonseca https://orcid.org/0000-0002-6716-905X

### Data availability

All of the data that support the findings of this study are available in the main text.

## ﻿Appendix 1

**Table A1.** Green Spaces (UGS) per district (“localidades”). In some instances, the park names as indicated on the collection labels differ from the official names, which are here provided in parentheses. See glossary of terms below, compilated from: Alcaldía Mayor de Bogotá 2021, 2022, IDRD 2023a, 2023b.

Neighbourhood / UGS	Identifier	Park level and typology	Park classification
**Antonio Nariño**			
Parque Ciudad Jardín	15-027	Structuring (sport)	Zonal
**Chapinero**			
Parque El Chicó	02-097	Proximity	Pocket
Parque El Virrey	02-014	Structuring (contemplative)	Zonal
Sendero Quebrada La Vieja	–	Protected area	–
**Engativá**			
Jardín Botánico de Bogotá	10-291	Structuring (contemplative)	Metropolitan
**Rafael Uribe Uribe**			
Parque Palermo Sur	18-035	Proximity	Neighbourhood
**San Cristóbal**			
Parque La Victoria	04-122	Structuring (sport)	Zonal
Parque Primero de Mayo (Deportivo Primero de Mayo)	04-196	Structuring (sport)	Metropolitan
Parque San Cristóbal	04-127	Structuring (cultural)	Metropolitan
Parque Villa de los Alpes	04-075	Structuring (sport)	Zonal
**Santa Fe**			
Parque La Independencia (Independencia-Bicentenario)	03-039	Structuring (cultural)	Metropolitan
Parque Nacional	03-035	Structuring (cultural)	Metropolitan
Parque Ilarco	11-007	Proximity	Neighbourhood
Parque Tercer Milenio	03-085	Structuring (cultural)	Metropolitan
Universidad Distrital (Pueblo Viejo)	–	Structuring (NIA)	Zonal
**Suba**			
Cerro La Conejera	–	Protected area	Ecological
Parque Canal Molinos (Parque La Alhambra Sector Sur)	11-097	Proximity	Neighbourhood
Vacant lot	–		
**Usaquén**			
CAI Santa Barbara (Urbanización Santa Bárbara Primer Sector)	01-198	Proximity	Neighbourhood
Parque Altablanca	01-075	Structuring (sport)	Zonal
Parque Belmira	01-187	Proximity	Neighbourhood
Parque Cabañas del Norte	01-244	Proximity	Neighbourhood
Parque CAI Lisboa (Urbanización Ginebra Norte)	01-120	Proximity	Neighbourhood
Parque Cedro Madeira (El Cedro Maderia)	01-250	Proximity	Neighbourhood
Parque Contador Norte	01-083	Proximity	Neighbourhood
Parque Ginebra-Bella Suiza	01-106	Proximity	Neighbourhood
Parque La Francia (Urbanización Los Molinos)	01-118	Proximity	Neighbourhood
Parque La Vida	01-012	Structuring (recreational)	Zonal
Parque Nueva Autopista	01-064	Structuring (contemplative)	Zonal
Parque Usaquén 2 (Santa Barbara Primer Sector)	01-087	Proximity	Neighbourhood
**Usme**			
Parque Chuniza-Famaco (Famaco)	05-086	Structuring (cultural)	Zonal
Parque La Andrea	05-004	Structuring (sport)	Zonal
Parque Virrey Sur	05-016	Structuring (sport)	Zonal

**Glossary**

**Contemplative**: Spaces designed to promote the richness and diversity of vegetation cover for environmental enjoyment and low-impact human activities. They focus on a contemplative and educational relationship achieved through both permanence and travel. Their main spatial design component is ecological.

**Cultural**: Spaces designed to serve as meeting places, promoting permanence for the development of civic or cultural activities and outdoor events that highlight cultural values, traditions, and collective memory. The design can incorporate various care and social services, with permanence as the main spatial design component.

**Ecological park**: Parks that due to their high scenic and/or biological value, as well as their location and accessibility, are intended for the preservation, restoration, and sustainable ecological use of their biophysical elements for environmental education and passive recreation.

**Metropolitan park**: Parks covering an area of more than 10 hectares, designated for the development of both active and passive recreational uses, aiming to generate landscape and environmental values. The influence of these spaces extends across the entire territory of the city.

**Neighbourhood park**: Parks with an area of less than one hectare, designed for the recreation, meeting, and integration of the community, addressing the specific needs of the neighbourhoods.

**Pocket park**: Neighbourhood-type parks but with an area of less than 0.1 hectares, intended primarily for the recreation of children and senior citizens.

**Protected area**: Spaces with unique value for the natural heritage of the Capital District, ecosystems, biodiversity conservation, and the evolution of culture in the area.

**Proximity space**: Spaces mostly smaller than one hectare that offer a diverse range of leisure activities at a local scale.

**Recreational**: Spaces designed to provide facilities for the development of recreational activities, promoting relationships between individuals, the development of skills, and engagement in both free and structured activities. Their main spatial design component is play.

**Sport**: Spaces designed to accommodate physical activities and sports practice at different levels, including recreational, training, and competitive levels. The activities focus on the physical conditioning of different age groups, either individually or collectively. The main spatial design component of these spaces is sports.

**Structuring space**: Spaces larger than one hectare that provide a diverse range of leisure activities, supporting both regional and district scales. These spaces contribute not only to human interactions but also to environmental and ecosystemic connectivity.

**Zonal park**: Parks ranging from 1 to 10 hectares designed to fulfil the active recreational needs of a group of neighbourhoods and can accommodate specialized sport facilities.

**References**

Alcaldía Mayor de Bogotá (2021) Anexo 03. Inventario de espacio público peatonal y para el encuentro. In: Plan de Ordenamiento Territorial: Bogotá Reverdece 2022–2035. Alcaldía Mayor de Bogotá, Secretaría Distrital de Planeación, Bogotá, 200. https://www.sdp.gov.co/sites/default/files/anexo_03_inventario_espacio_publico_0.pdf [Accessed 24 Nov 2023]

Alcaldía Mayor de Bogotá (2022) Manual de espacio público. Alcaldía Mayor de Bogotá, Secretaría Distrital de Planeación, Bogotá, 476 pp. https://www.sdp.gov.co/sites/default/files/generales/mep_p1-mon.pdf [Accessed 24 Nov 2023]

IDRD (2023a) Buscador de parques. https://portalciudadano.idrd.gov.co/parques/buscar [Accessed 24 Nov 2023]

IDRD (2023b) Parques. Instituto Distrital de Recreación y Deporte. https://sim1.idrd.gov.co/parques-0 [Accessed 24 Nov 2023]
